# Repression of tick microRNA-133 induces organic anion transporting polypeptide expression critical for *Anaplasma phagocytophilum* survival in the vector and transmission to the vertebrate host

**DOI:** 10.1371/journal.pgen.1008856

**Published:** 2020-07-02

**Authors:** Ellango Ramasamy, Vikas Taank, John F Anderson, Hameeda Sultana, Girish Neelakanta

**Affiliations:** 1 Department of Biological Sciences, Old Dominion University, Norfolk, Virginia, United States of America; 2 Department of Entomology, Connecticut Agricultural Experiment Station, New Haven, Connecticut, United States of America; 3 Center for Molecular Medicine, Old Dominion University, Norfolk, Virginia, United States of America; University of Maryland School of Medicine, UNITED STATES

## Abstract

The microRNAs (miRNAs) are important regulators of gene expression. In this study, we provide evidence for the first time to show that rickettsial pathogen *Anaplasma phagocytophilum* infection results in the down-regulation of tick microRNA-133 (miR-133), to induce *Ixodes scapularis* organic anion transporting polypeptide (*isoatp4056*) gene expression critical for this bacterial survival in the vector and for its transmission to the vertebrate host. Transfection studies with recombinant constructs containing transcriptional fusions confirmed binding of miR-133 to *isoatp4056* mRNA. Treatment with miR-133 inhibitor resulted in increased bacterial burden and *isoatp4056* expression in ticks and tick cells. In contrast, treatment with miR-133 mimic or pre-mir-133 resulted in dramatic reduction in *isoatp4056* expression and bacterial burden in ticks and tick cells. Moreover, treatment of ticks with pre-mir-133 affected vector-mediated *A*. *phagocytophilum* infection of murine host. These results provide novel insights to understand impact of modulation of tick miRNAs on pathogen colonization in the vector and their transmission to infect the vertebrate host.

## Author summary

This study provides novel evidence that shows that down-regulation of arthropod microRNA-133 leading to enhanced expression of organic anion transporting polypeptide is not only critical for rickettsial pathogen *Anaplasma phagocytophilum* survival in ticks but also for this bacterial transmission from vector to the vertebrate host. Understanding how pathogens manipulate vector-signaling repertoire for their benefit would lead to the development of strategies to block their transmission from vector to the vertebrate host.

## Introduction

In the United States, black-legged tick *Ixodes scapularis* is the primary vector for several human pathogens that includes anaplasmosis agent *Anaplasma phagocytophilum*, Lyme disease agent *Borrelia burgdorferi*, babesiosis agent *Babesia microti*, Ehrlichiosis agent *Ehrlichia muris* subsp. *eauclairensis* subsp. nov., Tick encephalitis viral disease agent Powassan virus and tick-borne relapsing fever agent *Borrelia miyamotoi* [1–5]. Ticks ingest these pathogens upon feeding on an infected reservoir host. Humans are the accidental hosts that get diseases by the bite of an infected tick [1–4]. Recent disease-surveillance data shows dramatic increase in these tick-borne diseases [6–11]. The control strategies to treat these diseases or to prevent transmission of pathogens from ticks to the vertebrate host, including humans, are limited. Therefore, understanding molecular basis of interactions of tick-borne pathogens with their vector host remains important.

In humans, the rickettsial pathogen *A*. *phagocytophilum* has developed various strategies to infect and survive in neutrophils and other cells of hematopoietic origin [12–17]. Some of the recent studies, including our own, have highlighted importance of tick molecules in the colonization of *A*. *phagocytophilum* in *I*. *scapularis* ticks [18–27]. Our previous studies provided extensive evidences on *A*. *phagocytophilum*-mediated modulation of tick signaling repertoire involving organic anion transporting polypeptide (OATP), kynurenine aminotransferase (KAT), antifreeze glycoprotein (IAFGP), phosphorylated actin, P21-activated kinase (PAK), G-protein subunits beta and gamma, Phosphoinositide 3 kinases (PI3K), Src tyrosine kinase and transcription activator protein (AP-1) [23–27]. The OATPs are highly conserved among arthropods [28]. Human OATPs are reported to be localized on the cell surface and are involved in the uptake of several molecules including anions, signaling molecules and metabolites [26, 28–31]. Arthropod OATPs not only play important role in bacteria-tick interactions but also are critical in virus-tick interactions [26, 28]. In ticks, OATPs are expressed in various tissues, including salivary glands, midgut, synganglion, Malpighian tubules and ovaries [26, 32]. Our previous report noted that exogenous addition of tryptophan metabolite, xanthurenic acid, up-regulates *isoatp4056* expression and increases *A*. *phagocytophilum* multiplication in ticks and tick cells [26]. Our data suggested that arthropod OATP serves as a transporter for xanthurenic acid in ticks and tick cells [26].

Several studies have reported the use of proteomics and transcriptomics to identify molecules that show differential levels in pathogen-infected ticks in comparison to uninfected ticks [33–36]. However, studies that address how pathogens differentially modulate tick gene expression at the post-transcriptional level are limited. A group of small non-coding RNAs termed as microRNAs (miRNAs) are highly involved in the post-transcriptional regulation of genes in animals, arthropods, plants, and in some viruses [37–41]. Since the initial discovery of miRNA in *Caenorhabditis elegans* in 1993, more than 2,000 miRNAs are estimated to be present in humans and approximately 1,300 miRNAs have been identified in parasites [42, 43]. The number of newly identified miRNAs in various organisms is still increasing [44].

The miRNAs are first synthesized as long primary pri-miRNA by RNA polymerase II [45, 46]. The pri-miRNA is then cleaved into approximately 70–100 base pairs short precursor pre-miRNA by drosha and then exported into cytoplasm and processed by dicer into 20–26 bp miRNA duplex consisting of guide strand and a passenger strand [46, 47]. The passenger strand gets degraded leaving guide strand to become matured miRNA [47]. The matured miRNA organizes into RNA-induced silencing complex (RISC) consisting of Ago2 and other accessory proteins [37, 40]. The whole RISC complex containing guide strand will bind to target mRNA transcripts leading to degradation of mRNA or translation repression [37, 40]. Studies have also reported that binding of miRNA to mRNA could stabilize the transcript or enhance transcription and/or translation [48, 49]. Most of miRNAs bind 3’ UTR of the target genes [50]. However, recent studies demonstrated that miRNA could bind its target genes at 5’ UTR region or in the CDS part of the transcript [51, 52]. In addition, miRNA targets containing simultaneous 5’-UTR and 3’-UTR interaction sites have been reported [53]. The efficiency of miRNA binding to its target mRNA is determined at its 5’ end by the presence of 6–8 nucleotides termed as “seed region” [37, 40].

Several studies have identified miRNAs in various tick species that includes *I*. *ricinus*, *Rhipicephalus sanguineus*, *R*. *haemaphysaloides*, *R*. *microplus*, *Hyalomma anatolicum* and *Haemaphysalis longicornis* [54–59]. However, studies that characterize arthropod miRNAs that are important in the transmission of pathogens from tick vector to vertebrate host are lacking. In this study, we identified several microRNAs from *I*. *scapularis* genome and show specific role for miR-133 in *A*. *phagocytophilum* colonization and transmission from ticks to vertebrate host. We provide extensive molecular evidence for the first time to show how rickettsial pathogen infection modulates arthropod miR-133 to enhance tick organic anion transporting polypeptide, *isoatp4056*, expression critical for this bacterial colonization in ticks and for its transmission from vector to vertebrate host.

## Results

### Analysis of the expression stability of selected reference gene candidates for normalization of microRNA levels in *Ixodes scapularis*

**Fig 1:**
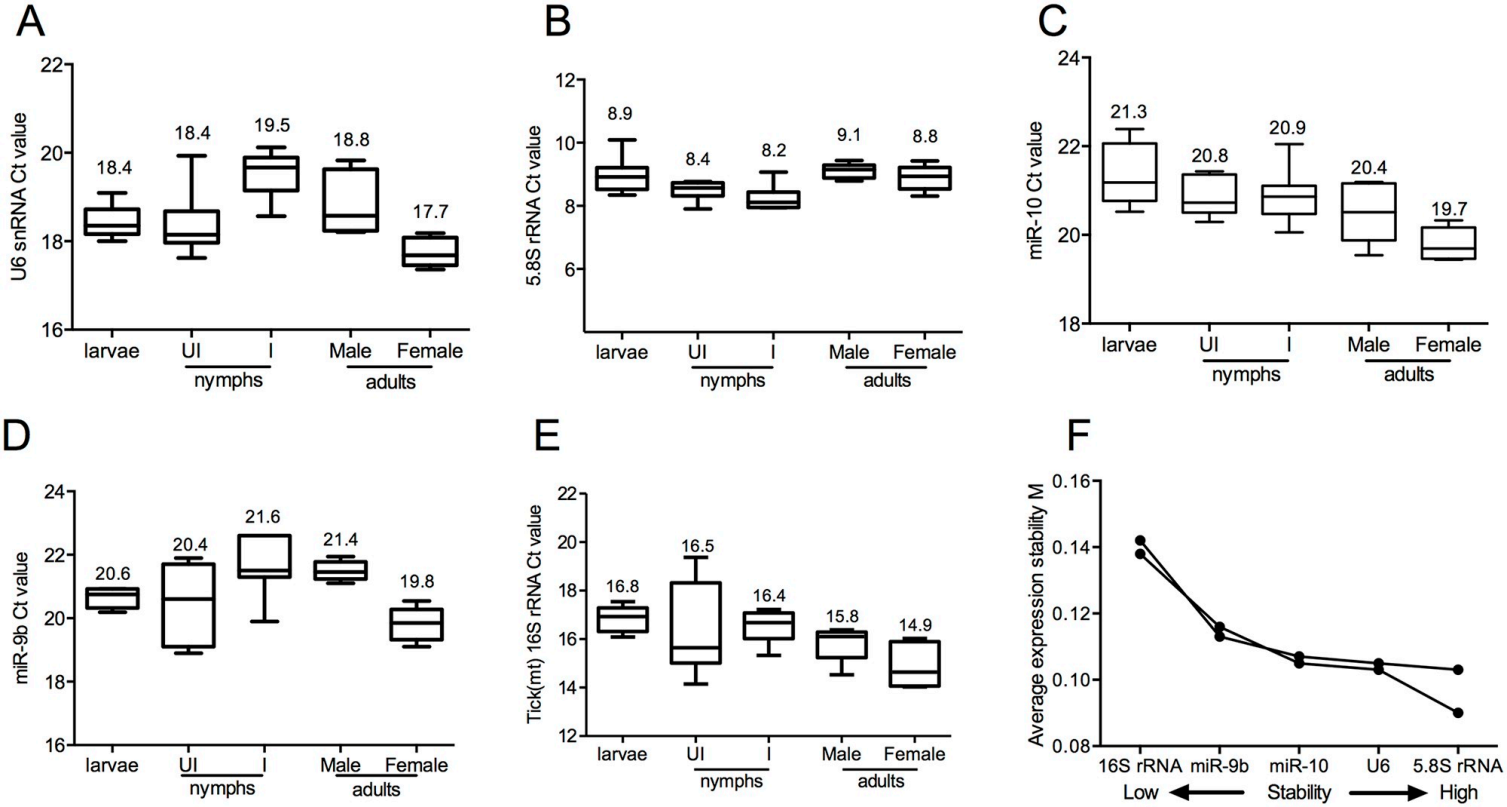
Expression and stability analysis of *I*. *scapularis* miRNA and other *I*. *scapularis* house-keeping genes in tick microRNA samples for reference gene selection. (A) QRT-PCR analysis showing levels of *I*. *scapularis* U6 snRNA (A), 5.8S rRNA (B), miR-10 (C), miR-9b (D) and tick mitochondrial (mt) 16s rRNA (E) in microRNA samples extracted from unfed uninfected larvae, unfed uninfected nymphs (UI), unfed *A*. *phagocytophilum*-infected nymphs (I), unfed uninfected adult male or female ticks. The box chart indicates the interquartile range. The lower and upper dashes depict the minimum and maximum values. The line across the box is the median. F) Stability report of all five reference genes investigated using GeNorm analysis is shown. Stability measure *M* for the candidate genes was calculated as the average pairwise variation for that gene with all other tested genes. The arrows indicate direction of stability range from low to high.

Quantitative real-time PCR (QRT-PCR) that requires normalization of miRNA levels to a reference gene is critical to ensure accuracy in quantification [60, 61]. We first carefully evaluated the stability in the expression pattern of reference genes using specific oligonucleotides (S1 Table) that can be considered to normalize miRNA levels in *I*. *scapularis* ticks. We considered five commonly analyzed reference genes to validate their expression stability in different developmental stages of ticks and in the presence of rickettsial pathogen *Anaplasma phagocytophilum*. The five house-keeping genes analyzed in this study includes small nuclear ribonucleoprotein U6 (snRNA U6) (Fig 1A), 5.8S ribosomal RNA (5.8S rRNA) (Fig 1B), *I*. *scapularis* miRNA 10 (miR-10) (Fig 1C), *I*. *scapularis* miRNA 9b (miR-9b) (Fig 1D) and *I*. *scapularis* mitochondrial 16S ribosomal RNA (16S rRNA) (Fig 1E). The Cycle threshold (Ct) values in QRT-PCR gave an overview of the variations in gene expression in the analyzed samples. Equal amounts of same cDNA prepared from equal amounts of RNA isolated from each group were used in this analysis. All selected reference genes had variable expression values in different developmental stages of *I*. *scapularis* and in the presence of *A*. *phagocytophilum* (Fig 1A–1E). The 5.8S rRNA (Fig 1B) had a highest expression (mean Ct = 8.2 to 9.1) and miR-9b (Fig 1D) had the lowest expression (mean Ct = 19.8 to 21.6) in tested conditions. In addition, 5.8S rRNA (Fig 1B) had the lowest standard deviation values in comparison to the other tested genes (Fig 1A, 1C, 1D and 1E). Furthermore, expression stability was evaluated by GeNorm by calculating stability measure *M* for the candidate genes as the average pairwise variation for that gene with all other tested genes as described [62]. The GeNorm analysis revealed high to low stability for the genes in the order *5*.*8S rRNA* = *U6* > *miR-10* > *miR-9b* > 16S rRNA (Fig 1F). Collectively, these results reveal that 5.8S rRNA with low M value and standard deviation is an ideal reference gene for normalization of miRNA levels in *I*. *scapularis* ticks.

### *Anaplasma phagocytophilum* infection results in the down-regulation of specific *I*. *scapularis* miRNAs

**Fig 2:**
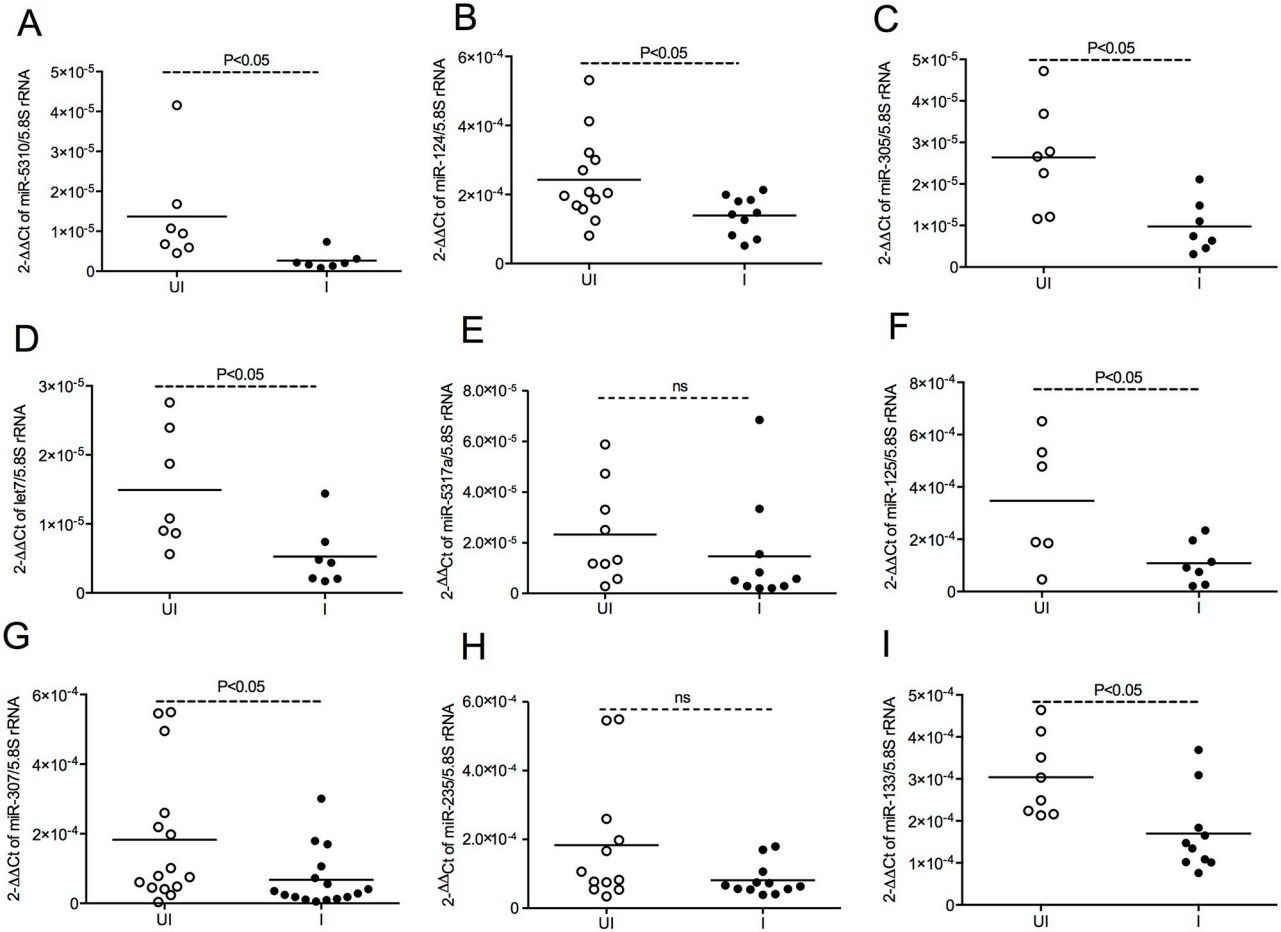
*A*. *phagocytophilum* differentially regulate some of the miRNAs in ticks. QRT-PCR analysis showing expression of miR-5310 (A), miR-124 (B), miR-305 (C), let7 (D), miR-5317a (E), miR-125 (F), miR-307 (G), miR-235 (H) and miR-133 (I) in unfed uninfected and *A*. *phagocytophilum-*infected nymphs. Open circle represents uninfected (UI) and closed circles represent infected (I) nymphs. Each circle represents miRNA samples generated from pool of three nymphs. Expression of miRNAs was normalized to tick 5.8S rRNA levels. P value from non-paired student’s t-test is shown. ns indicates not significant.

Analysis of *I*. *scapularis* genome (NCBI Bioproject PRJNA16232) with screening for all previous reported mature animal miRNA and precursor sequences at miRBase followed by BLASTn search revealed presence of several miRNAs including miR-5310, miR-124, miR-305, let7, miR-5317a, miR-125, miR-307, miR-235, and miR-133 (Fig 2, S1 Fig and S2 Table). The “Mfold” analysis revealed *I*. *scapularis* miRNA secondary structures and its energy of folding value (S1 Fig). Previous studies have indicated modulation of several signaling events in *I*. *scapularis* ticks upon *A*. *phagocytophilum* [23–27]. We therefore tested if miRNA expression is also modulated upon *A*. *phagocytophilum* infection in these ticks. QRT-PCR analysis with miRNAs isolated from unfed uninfected or *A*. *phagocytophilum*-infected nymphal ticks revealed that the rickettsial pathogen significantly (P<0.05) down-regulates miR-5310 (Fig 2A), miR-124 (Fig 2B), miR-305 (Fig 2C), let 7 (Fig 2D), miR-125 (Fig 2F), miR-307 (Fig 2G) and miR-133 (Fig 2I). No significant difference was noted in the expression of miR-5317a (Fig 2E), miR-235 (Fig 2H), miR-9b (S2A Fig) and miR-10 (S2B Fig) between unfed uninfected and *A*. *phagocytophilum*-infected nymphal ticks. Collectively, the data from Figs 1 and 2 shows that upon *A*. *phagocytophilum* infection expression of specific *I*. *scapularis* miRNAs are down-regulated.

### Target site prediction for *I*. *scapularis* miRNAs

**Fig 3:**
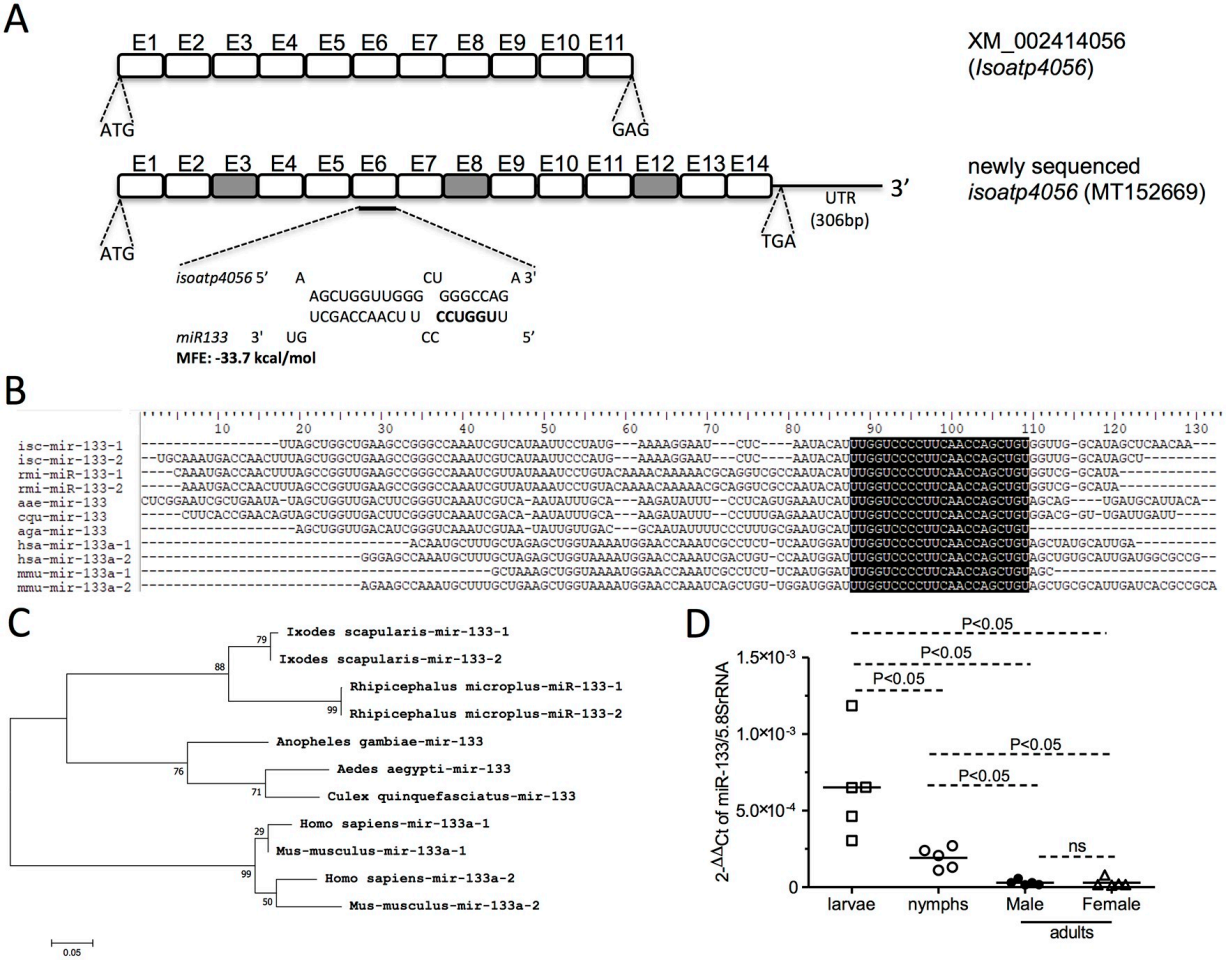
Bioinformatic analysis of *I*. *scapularis* pre-mir-133 sequence with other miRNA sequences. (A) Schematic representation of the complete *isoatp4056*-coding region with 306 bp of 3’ UTR region (GenBank acc. no. MT152669). Both start and stop codons are indicated in the newly sequenced *isoatp4056* with three new possible exons. The miR-133 binding site at exon 6 (E6) region of *isoatp4056* mRNA is shown. The miR-133 nucleobases shown in bold indicates the microRNA seed region. NCBI accession number for the previously reported *isoatp4056* transcript is provided. MFE indicates minimum energy of folding. (B) *I*. *scapularis* pre-mir-133 sequence alignment with other blood-feeding arthropods, human and mice shows no variations at mature sequence region (bases highlighted). (C) Phylogenetic analysis showing distribution of *I*. *scapularis* pre-mir-133 sequences with other pre-mir-133 sequences from blood-feeding arthropods, human & mice. GenBank or miRBase accession numbers are provided in the methods section and discussed in results section (D) QRT-PCR analysis showing expression of *I*. *scapularis* mature miR-133 levels at different tick developmental stages. Each square, open circle, closed circle and triangle represents data obtained from one pool of unfed uninfected tick samples. For larvae, data was obtained from pool of 5 ticks and for nymphs data was obtained from pool of 3 ticks. For adult male and females, data was obtained from an individual tick. Expression of miR-133 was normalized to tick 5.8S rRNA levels. P value from non-paired student’s t-test is shown. ns indicates not significant.

The down-regulation of several miRNAs in the presence of *A*. *phagocytophilum* suggests that infection with this bacterium results in sequestration of most of these miRNAs to target specific genes in *I*. *scapularis* ticks. Our previous studies reported that *A*. *phagocytophilum* modulates organic anion transporting polypeptide, IsOATP4056 and kynurenine amino transferase (KAT) signaling for its survival in ticks [23, 26]. Due to availability of only partial coding sequence of *isoatp4056* (GenBank acc. no. XM_002414056.1), we amplified full-length mRNA along with 3’ UTR region. The whole coding sequence (CDS) along with 3’UTR region was sequenced (GenBank acc. no. MT152669). The nucleotide sequencing analysis revealed identification of three possible new exons (Fig 3A), in addition to the 11 exons previously reported in GenBank (Acc. no. XM_002414056.1) database. The newly sequenced *isoatp4056* CDS showed presence of additional sequences at three different regions in XM_002414056.1. These additional sequences were not found in one stretch in the newly sequenced *isoatp4056* CDS. A putative stop codon and 3’ UTR region corresponding to 306 base pair (bp) was identified in the newly sequenced *isoatp4056* gene (Fig 3A). Analysis of target sites for all the newly identified and known miRNAs analyzed in this study on *isoatp4056* CDS and 3’ UTR region revealed miR-133-binding site on the coding sequence region (Fig 3A).

### miR-133 is developmentally regulated in *Ixodes scapularis* ticks

Screening of *I*. *scapularis* genome and miRBase revealed presence of two pre-mir-133 sequences with a nucleotide change between the two sequences (miRBase acc. no. MI0012266 and GenBank acc. DS613658). CLUSTALW alignment of both *I*. *scapularis* pre-mir-133 sequences revealed no variations in the mature miR-133 region (Fig 3B). In addition, CLUSTALW alignment of *I*. *scapularis* pre-mir-133 sequences with human miR-133a-1 and miR-133a-2 (miRBase acc. nos. MI0000450, MI0000451), mice miR-133a-1 and miR-133a-2 (miRBase acc. nos. MI0000159, MI0000820) and other arthropod vector miR-133 sequences including *Rhipicephalus microplus* (GenBank acc. nos. LYUQ01278569, LYUQ01042747), *Aedes aegypti* (miRBase acc. no. MI0013482), *Culex quinquefasciatus* (miRBase acc. no. MI0013625) and *Anopheles gambiae* (miRBase acc. no. MI0001606) revealed no variations in the mature miR-133 region (Fig 3B). Phylogenetic tree generated from the alignment revealed that all pre-mir-133 sequences from ticks fall within the same clade (Fig 3C) suggesting that they might be regulated in a similar way to form mature miRNA. The pre-mir-133 from mosquitoes forms a different clade (Fig 3C). Human and mice pre-mir-133 sequences fall within the same clade but formed a different clade when compared to ticks and mosquitoes (Fig 3C). Analysis of mature miR-133 region revealed no variations in any of the sequences analyzed in this study (Fig 3C). MicroRNAs play important roles in the developmental process of various organisms [63]. We therefore analyzed expression of miR-133 in different tick developmental stages. QRT-PCR analysis revealed that mature miR-133 expression is developmentally regulated in *I*. *scapularis* ticks (Fig 3D). Unfed larval ticks expressed significantly (P<0.05) higher level of miR-133 in comparison to the levels noted in unfed nymphal and adult ticks (Fig 3D). Expression of miR-133 was significantly (P<0.05) higher in nymphal ticks in comparison to the levels noted in adult male and female ticks (Fig 3D). No significant differences in the miR-133 levels were noted between adult male and female ticks (Fig 3D). Furthermore, we noted significantly decreased (P<0.05) expression of miR-133 (S2C Fig) and increased bacterial loads (S2D Fig) in larval ticks fed on *A*. *phagocytophilum*-infected mice in comparison to the relative levels (S2C and S2D Fig) noted in molted unfed *A*. *phagocytophilum*-infected nymphal ticks. Collectively, these results not only indicate that expression of miR-133 is developmentally regulated but also shows that decreased levels of miR-133 expression leads to increased *A*. *phagocytophilum* loads in ticks.

### miR-133 targets *Ixodes scapularis* organic anion transporting polypeptide (*isoatp4056*) mRNA

**Fig 4:**
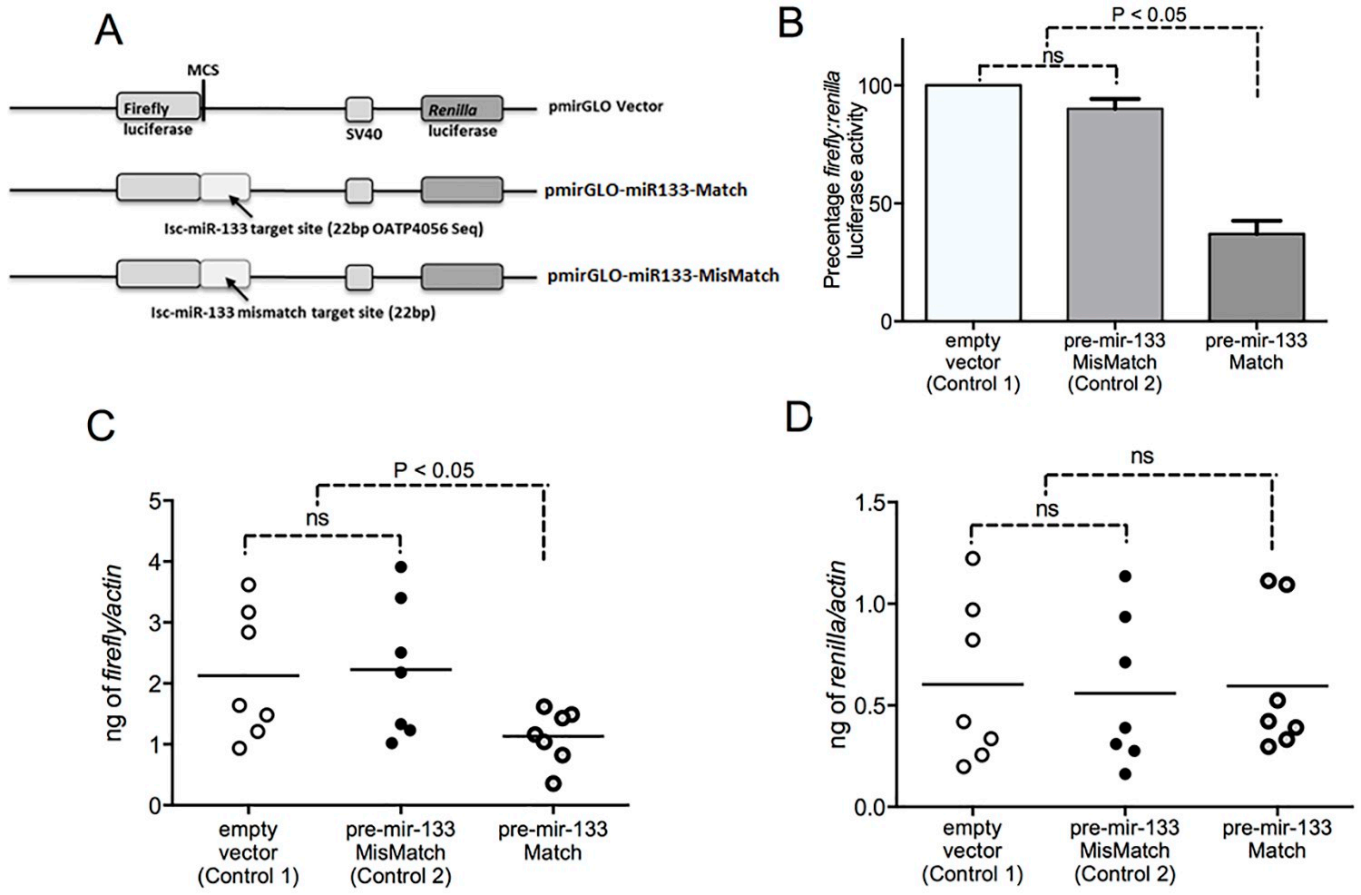
Dual luciferase assay confirms *I*. *scapularis* miR-133 binding site on *isoatp4056* mRNA. (A) Schematic representation of miR-133 constructs used in dual luciferase assay is shown. The 22 bp *isoatp4056* target site for miR-133-binding (pmirGLO-miR-133 match) or a 22 bp mismatch sequence (pmirGLO-miR-133-mismatch) carrying a base change at the miR-133 seed region binding are transcriptionally fused to Firefly transcript. Both pmirGLO empty vector and pmirGLO-mismatch constructs was used as controls (control 1 and control 2). MCS represent multiple cloning site and Isc represents *I*. *scapularis*. (B) Dual Luciferase activity measurement from the cell culture supernatants of ISE6 tick cells transfected with pmirGLO-miR-133-match or pmirGLO empty vector (control 1) or pmirGLO-miR-133-mismatch (control 2) is shown. Bar graphs for ISE6 cells transfected with pmirGLO-miR-133-match represents percentage of firefly/renilla luciferase activity compared to activity noted in empty vector (control 1) or pmirGLO-miR-133-mismatch (control 2) transfected cells. QRT-PCR analysis showing levels of Firefly (C) or Renilla (D) luciferase transcripts in ISE6 cells transfected with constructs shown in panel A. Expression of Firefly or Renilla transcripts analyzed in this data was normalized to tick beta-actin levels. Statistical analysis was performed using non-paired student’s t-test and P value is shown. ns indicates not significant.

We first tested if tick cells express miR-133. QRT-PCR analysis revealed that ISE6 tick cells do not express detectable levels of miR-133 (S3 Fig). Therefore, we generated synthetic oligonucleotide that can be used to synthesize pre-mir-133 (S4 Fig). The pre-mir-133 is a precursor form of microRNA that exists in secondary hairpin structure. Upon transfection into tick cells, pre-mir-133 would lead to the processing of mature miR-133. To test if miR-133 binds to the predicted target site on *isoatp4056* mRNA, we inserted miR-133 target site from *isoatp4056* sequence at the 3’ end of firefly luciferase gene in pmirGLO vector (Fig 4A). In addition, a fragment containing a mismatch in the *isoatp4056* miR-133 target site (miR133-mismatch) was cloned in a similar manner (Fig 4A). In miR-133-mismatch, seed region nucleobase was changed in *isoatp4056* target binding sequence. This will lead to mismatched base-pairing with miR-133. Tick cells were first treated with synthetic mir-133 and then transfected independently with three constructs (pmirGLO-*isoatp4056*-miR-133 target site sequence, pmirGLO-mismatch-miR-133 target site sequence and pmirGLO empty vector). Luciferase assay measurements revealed that tick cells carrying pre-mir-133 and transfected with pmirGLO- *isoatp4056*-miR-133 target site had significantly decreased (P<0.05) level of luciferase activity in comparison to the levels noted in tick cells with pre-mir-133 and transfected with constructs carrying either mismatch sequence or a empty vector controls (Fig 4B). No significant differences were noted between tick cells with pre-mir-133 and transfected with constructs carrying mismatch sequence or empty vector (Fig 4B). Furthermore, QRT-PCR analysis revealed that firefly luciferase transcripts were significantly decreased (P<0.05) in tick cells carrying pre-mir-133 and transfected with pmirGLO- *isoatp4056*-miR-133 in comparison to the levels noted in controls (Fig 4C). As expected, no significant differences in renilla luciferase expression was noted between any of these groups (Fig 4D). These results confirm that miR-133 binds *isoatp4056* CDS sequence. In addition, this assay provides important method to assess and confirm any miRNA binding to its mRNA targets in arthropods.

### Exogenous treatment with miR-133 affects *isaotp4056* expression and *Anaplasma phagocytophilum* survival in ticks

**Fig 5:**
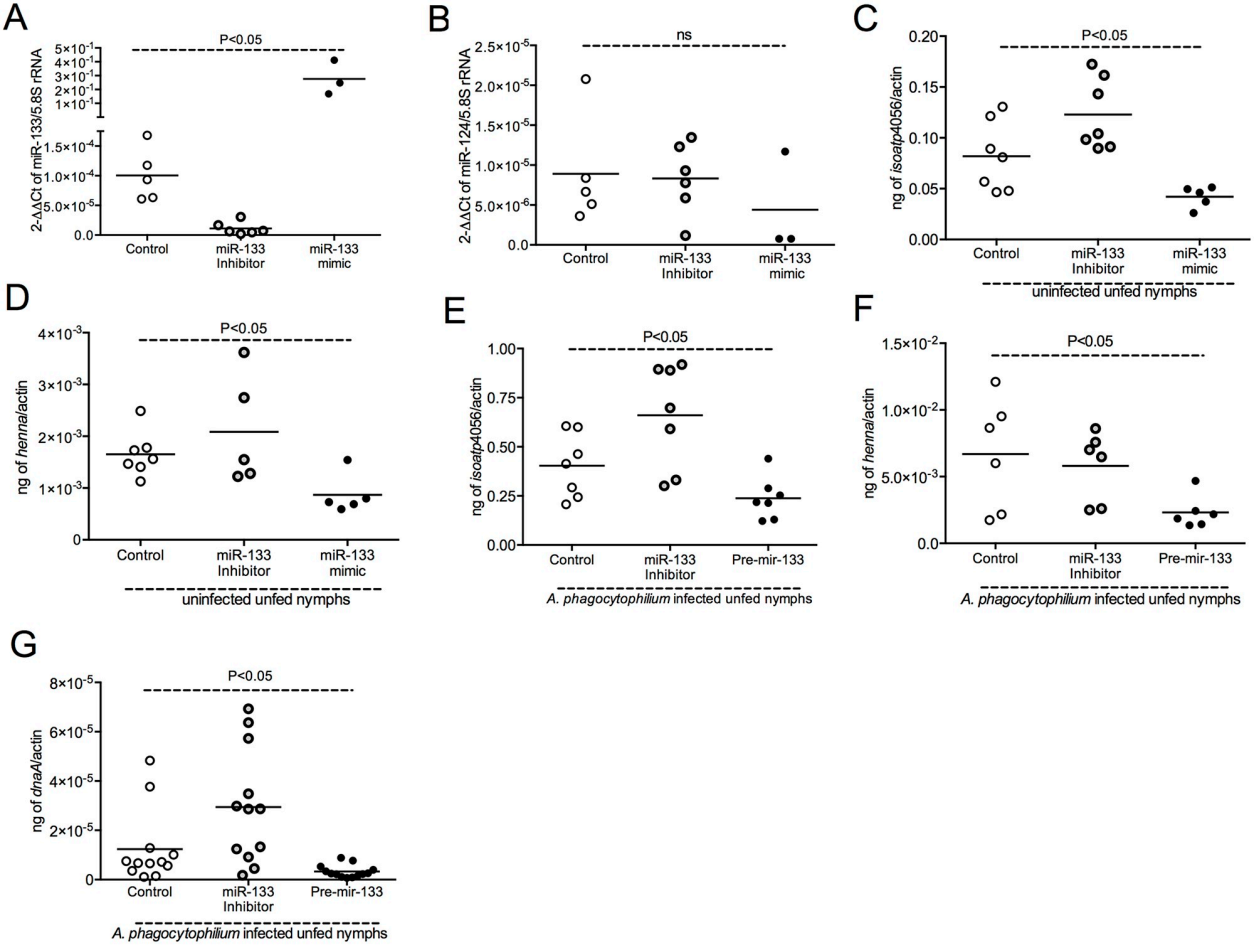
Treatment of ticks with miR-133 mimic or pre-mir-133 inhibits *A*. *phagocytophilum* survival in the vector. QRT-PCR data showing levels of miR-133 (A), mir-124 (B), *isoatp4056* (C, E) and *henna* (D, F) transcripts in unfed uninfected nymphs (A-D) or unfed *A*. *phagocytophilum*-infected (I) nymphs (E, F) after 48 h post-treatment with miR-133 mimic/pre miR-133 or miR-133-specific inhibitor or mock. The miRNA levels are normalized to tick 5.8S rRNA levels and *isoatp4056* or *henna* transcripts were normalized to tick beta-actin levels. (G) *A*. *phagocytophilum* loads in pre-mir-133 or miR-133 inhibitor-treated nymphal ticks at 48 h post treatment is shown. Each circle in panel A and B indicates data from pool of three unfed nymphs and each circle in panel C-G indicates data obtained from individual tick. Bacterial burden was evaluated by analyzing bacterial *dnaA* levels normalized to tick beta-actin DNA levels. In all panels, open circle indicates data from mock treated ticks, black circle indicates data from pre-mir-133/miR-133 mimic-treated ticks and grey circle indicates data from miR-133-inhibitor-treated ticks. Statistical analysis was performed using ANOVA and P value is shown. ns indicates not significant.

To analyze the effect of miR-133 overexpression or silencing on *isoatp4056* mRNA levels, uninfected nymphal ticks were microinjected with miR-133 mimic (synthetically generated mature form of miR-133) and miR-133 inhibitor (synthetically generated single stranded modified RNA that specifically inhibits endogenous miRNA function), respectively. QRT-PCR analysis revealed that ticks microinjected with miR-133 mimic had significantly increased (P<0.05) levels of mature miR-133 in comparison to ticks microinjected with miR-133 inhibitor or mock control (Fig 5A). The level of miR-133 was significantly (P<0.05) reduced in miR-133 inhibitor-treated ticks in comparison to the levels noted in control ticks (Fig 5A). No significant (P>0.05) difference in the miR-124 levels was noted between any of these groups (Fig 5B) providing specificity for the results obtained in Fig 5A. QRT-PCR analysis revealed significantly (P<0.05) reduced levels of miR-133 target gene, *isoatp4056*, in miR-133-mimic-treated ticks in comparison to the mock-treated or miR-133 inhibitor-treated groups (Fig 5C). Significantly (P<0.05) higher levels of *isoatp4056* transcripts were noted in miR-133 inhibitor-treated ticks in comparison to the levels noted in control ticks (Fig 5C). Treatment of ticks with pre-mir-133 also resulted in significant reduction in *isaoatp4056* transcripts (S5A Fig).

A recent study reported that miR-133 targets *henna*, a gene critical in dopamine secretion pathway in Locusts [64]. We noted no significant (P>0.05) differences in *henna* transcript levels between uninfected and *A*. *phagocytophilum*-infected ticks (S5B Fig). However, we considered *henna* as a positive control for validating miR-133 target binding. We analyzed *I*. *scapularis henna* transcripts in miR-133 mimic- and miR-133 inhibitor-treated ticks. QRT-PCR analysis revealed significant (P<0.05) reduced levels of *henna* transcripts in miR-133 mimic-treated ticks in comparison to miR-133-inhibitor- or control ticks (Fig 5D). Similar observation in *isoatp4056* (Fig 5E) and *henna* (Fig 5F) expression was noted in pre-mir-133- or miR-133-inhibitor-treated *A*. *phagocytophilum*-infected ticks. Furthermore, QRT-PCR revealed significant (P<0.05) reduction in *A*. *phagocytophilum* burden in pre-mir-133-treated ticks in comparison to miR-133 inhibitor-treated or control ticks (Fig 5G). In contrast, significantly (P<0.05) increased *A*. *phagocytophilum* burden was evident in miR-133 inhibitor-treated ticks in comparison to the burden noted in control ticks (Fig 5G). These results indicate that down-regulation of miR-133 results in increased *isoatp4056* expression important for *A*. *phagocytophilum* replication in ticks.

### Exogenous treatment with pre-mir-133 is not toxic to tick cells but affects *isoatp4056* expression and *Anaplasma phagocytophilum* survival in these cells

**Fig 6:**
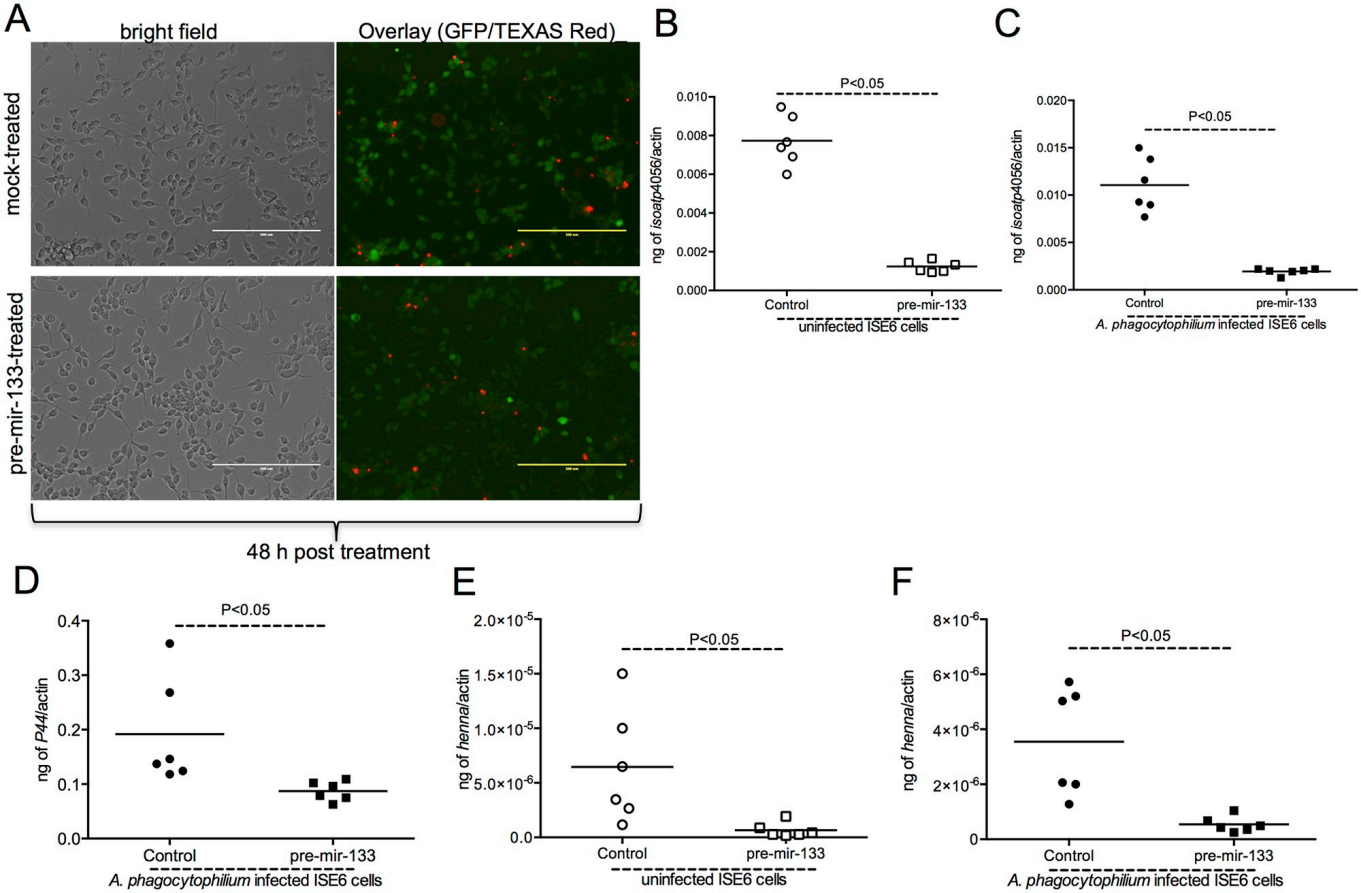
Treatment with pre-mir-133 is not toxic to tick cells but inhibits *isoatp4056* expression and affects *A*. *phagocytophilum* survival in these cells. Phase contrast or fluorescent microscopic images showing live (green) and dead (red) tick cells. Tick cells were treated with pre-mir-133 for 48 h and processed for staining using Live/Dead staining kit followed by imaging using EVOS fluorescent microscope. Scale bar indicates 200 μm. QRT-PCR data showing levels of *isoatp4056* transcripts (B, C), bacterial burden (D) and *henna* transcripts (E, F) in uninfected (B, E) or *A*. *phagocytophilum*-infected (C, D, F) tick cells after 48 h post-treatment with pre miR-133 or mock is shown. The mRNA levels of *isoatp4056* or *henna* or *A*. *phagocytophilum* P44 levels were normalized to tick beta-actin. Each circle and square all panels represent data from one independent culture plate well. Open circle/square indicates data from uninfected ticks and closed circle/square indicates data from *A*. *phagocytophilum*-infected ticks. Statistical analysis was performed using Student’s t test and P value is shown.

The observation of reduced bacterial burden in ticks upon exogenous treatment with pre-mir-133, promoted us to test this effect in tick cells *in vitro*. We first tested if treatment of tick cells with pre-miR-133 is toxic to tick cells. *In vitro* live/dead assays performed with uninfected tick cells at 24 and 48 h post-treatment time points revealed no significant difference in number of dead cells between mock or pre-miR-133-treated cells (Fig 6A, and S6A and S6B Fig). However, treatment of uninfected ISE6 cells with pre-mir-133 resulted in dramatic reduction (P<0.05) of *isoatp4056* transcripts (Fig 6B). Similarly, ISE6 cells treated with pre-mir-133 followed by *A*. *phagocytophilum* infection resulted in significant reduction in *isoatp4056* transcripts (Fig 6C) and bacterial burden (Fig 6D). The positive control gene, *henna*, transcripts were also significantly reduced upon pre-mir-133 treatment in both uninfected (Fig 6E) and *A*. *phagocytophilum*-infected (Fig 6F) tick cells. These results validate *in vivo* results that show the impact of down-regulation of miR-133 to enhance *isoatp4056* expression critical for *A*. *phagocytophilum* colonization in ticks and tick cells.

### Exogenous treatment with miR-133 does not affect tick engorgement weights

**Fig 7:**
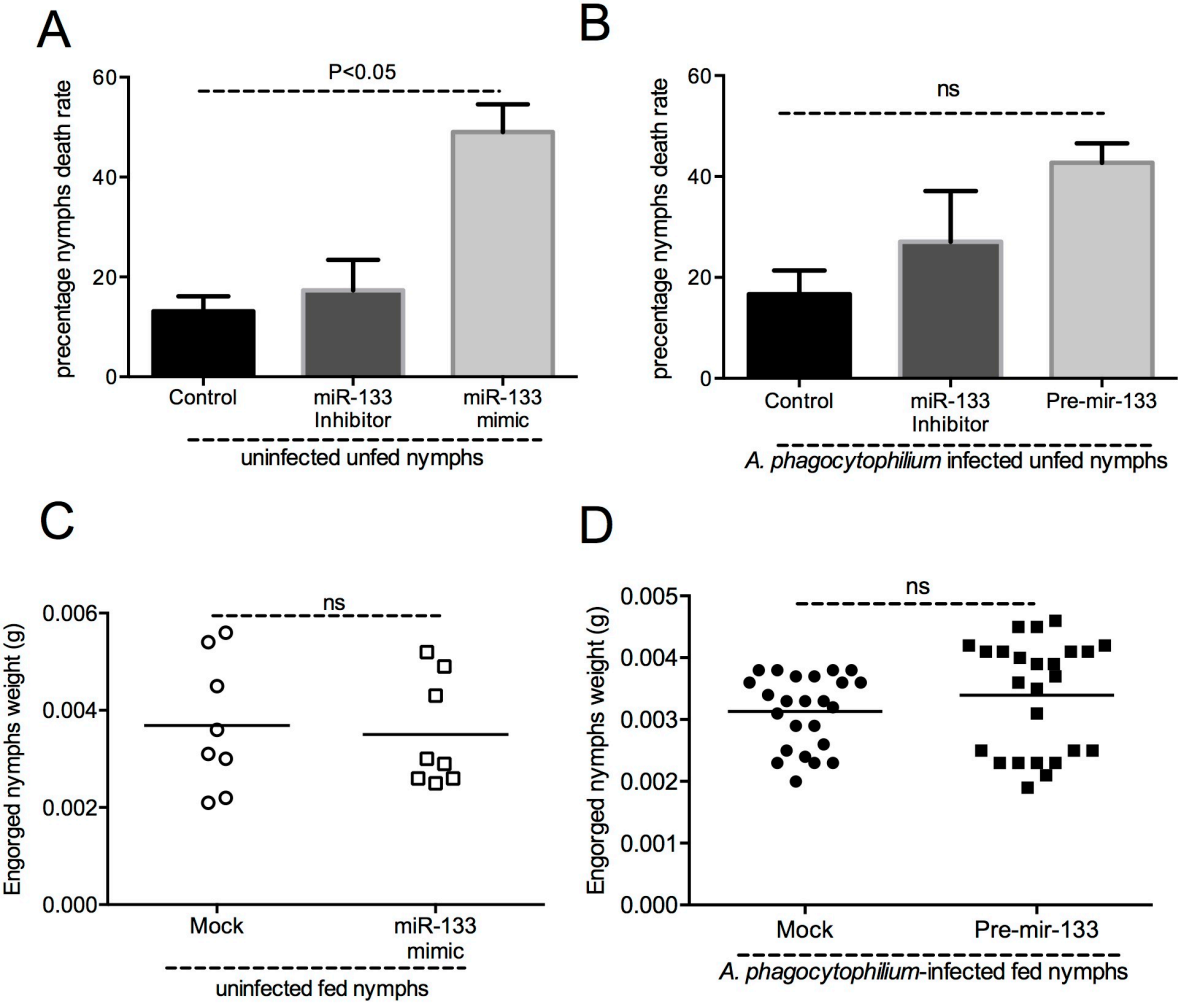
Treatment of ticks with miR-133 mimic or pre-mir-133 does not affect tick engorgement. Percentage death rate of unfed uninfected (A) or *A*. *phagocytophilum*-infected (B) nymphs at 48 h post-treatment with miR-133 mimic or pre miR-133 or miR-133 inhibitor is shown in comparison to control-treated ticks. Engorgement weights (in grams) of uninfected (C) or *A*. *phagocytophilum*-infected (D) mock-treated or miR-133- or pre-mir-133-treated is shown. ANOVA was used to calculate statistical significance in A and B and Student’s t test was used to calculate statistical significance in C and D. P values are shown. ns indicates not significant.

We then evaluated effect of exogenous treatment of miR-133 on unfed tick survival. Uninfected or *A*. *phagocytophilum*-infected unfed ticks were microinjected with miR-133 mimic or miR-133 inhibitor and then analyzed for viability at 48 h post injection. Analysis of the death rate indicated that uninfected miR133-mimic-treated unfed nymphal ticks showed significantly (P<0.05) higher death rate (40–50%) in comparison to miR-133 inhibitor- and mock-treated control (10–20%) ticks (Fig 7A). Even though the trend looks similar to uninfected ticks, no significant difference (P>0.05) in the death rate was noted between *A*. *phagocytophilum*-infected pre-mir-133-treated ticks in comparison to miR-133 inhibitor- and mock-treated control ticks (Fig 7B). We further tested if exogenous treatment of miR-133 affects tick feeding. However, the effect of exogenous treatment of miR-133 mimic or pre-miR-133 did not affected engorgement weights of uninfected or *A*. *phagocytophilum* ticks (Fig 7C and 7D), respectively. The engorgement weights of miR-133 mimic/pre-mir-133-treated uninfected or *A*. *phagocytophilum*-infected ticks were not significantly different from mock-treated ticks (Fig 7C and 7D). These results suggest that exogenous treatment of miR-133 do not affect tick fitness during feeding.

### Exogenous treatment with pre-mir-133 affects *isoatp4056* levels and transmission of *A*. *phagocytophilum* from ticks to vertebrate host

**Fig 8:**
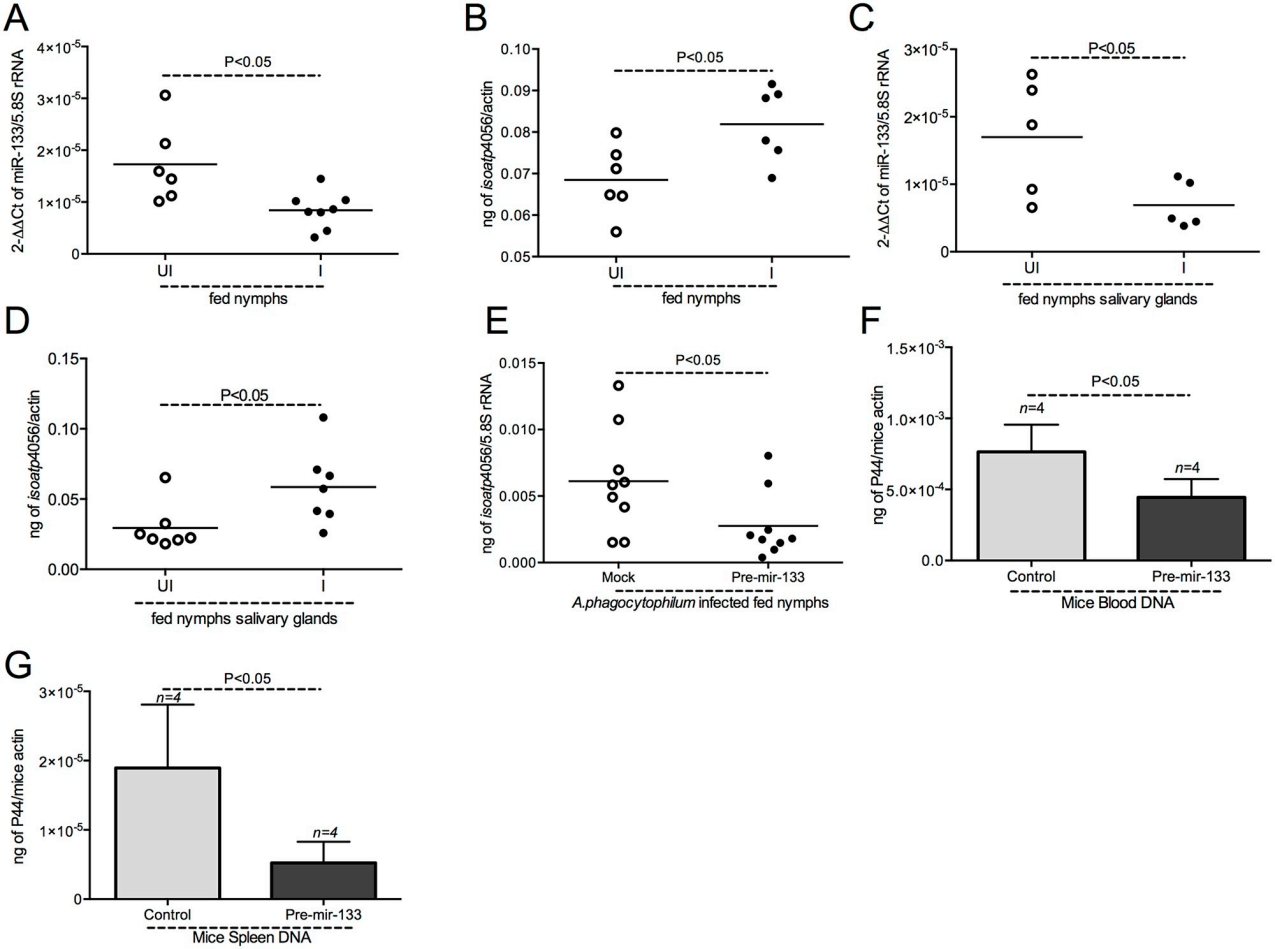
Treatment of ticks with pre-mir-133 affects *A*. *phagocytophilum* transmission from ticks to mice. QRT-PCR analysis showing levels of miR-133 (A, C) and *isoatp4056* (B, D) in uninfected (UI) or *A*. *phagocytophilum*-infected (I) nymphal whole ticks (A, B) or salivary glands (C, D) after feeding on naïve mice. E) QRT-PCR analysis showing levels of *isoatp4056* in *A*. *phagocytophilum*-infected fed nymphal ticks injected with mock or pre-mir-133. Open circles indicate data from uninfected ticks (UI) and closed circle indicates data from *A*. *phagocytophilum*-infected ticks. Each circle represents one tick. The miR-133 levels were normalized to tick 5.8S rRNA levels and *isoatp4056* transcript levels were normalized to tick beta-actin or 5.8S rRNA levels. Bacterial burden in murine blood (F) or spleen tissue (G) collected from mice infested with *A*. *phagocytophilum*-infected nymphal ticks injected with pre-mir-133 or mock is shown. *A*. *phagocytophilum p44* gene DNA levels were normalized to mice beta-actin levels. n indicates number of mice per treatment. Statistical analysis was performed using Student’s t test and P value is shown.

We then analyzed if *A*. *phagocytophilum* infection modulates miR-133 levels during its transmission from ticks to murine host. QRT-PCR analysis revealed that miR-133 was significantly (P<0.05) down-regulated in nymphal ticks during *A*. *phagocytophilum* transmission from ticks to mice (Fig 8A). We therefore, analyzed miR-133 target gene (*isoatp4056*) levels in ticks during transmission of *A*. *phagocytophilum*. QRT-PCR analysis showed that *isoatp4056* levels were significantly upregulated in *A*. *phagocytophilum*-infected ticks in comparison to the levels noted in uninfected ticks after feeding on naïve mice (Fig 8B). QRT-PCR analysis also showed that the levels of miR-133 were significantly down-regulated (Fig 8C and S7A Fig) and levels of *isaotp4056* were significantly up-regulated (Fig 8D and S7B Fig) in both salivary glands (Fig 8A and 8D) and gut tissues (S7A and S7B Fig) isolated from *A*. *phagocytophilum*-infected ticks in comparison to the levels noted in salivary glands and gut tissues isolated from uninfected ticks after feeding on naïve mice.

To test if miR-133-mediated *isoatp4056* regulation affects bacterial transmission from ticks to vertebrate host, *A*. *phagocytophilum-*infected unfed nymphal ticks were microinjected with pre-mir-133 or mock control and fed on naïve mice. QRT-PCR results showed significant reduction in *isoatp4056* transcripts in pre-mir-133-microinjected fed ticks in comparison to mock-injected control ticks (Fig 8C). Measurement of bacterial burden revealed significant (P<0.05) reduction in *A*. *phagocytophilum* loads in blood (Fig 8D) and spleen tissue (Fig 8E) collected from mice infested with pre-mir-133-injected ticks in comparison to the loads noted in tissues collected from mice infested with mock-injected control ticks. Collectively, these results elucidate that *A*. *phagocytophilum* infection results in the modulation of miR-133 to enhance *isoatp4056* expression critical for this bacterial transmission from ticks to vertebrate host.

## Discussion

The studies in the identification and characterization of tick molecular determinants involved in vector-pathogen interactions could lead to the development of better strategies to treat/control tick-borne diseases [65]. The requirement of arthropod OATPs for both bacteria and viral pathogens to colonize and survive in ticks [26, 28] suggests that these family of molecules could be considered as ideal therapeutic candidates for the development of anti-vector vaccine(s) [65]. Our previous studies elucidated that *A*. *phagocytophilum* modulates arthropod signaling via activator protein-1 (AP-1) and phosphorylated actin at the transcription-initiation level [23, 25]. In this study, we provide novel findings to understand tick molecular signaling at post-transcriptional level important for *A*. *phagocytophilum* survival in the vector host and during its transmission to the vertebrate host.

The previously reported *isoatp4056* GenBank sequence (acc. no. XM_002414056.1) had missing exons in the coding sequence and had limited information on 3’ UTR. In this study, the newly sequenced *isoatp4056* gene (GenBank acc. no. MT152669) revealed presence of possible additional exons and provided more information on the 3’ UTR. The binding of miR-133 on CDS of Locusts *henna* gene is reported [64]. Therefore, the observation of the binding of miR-133 at its target site on *isoatp4056* CDS but not on 3’ UTR is not surprising. Our study also revealed that treatment of ticks and tick cells with miR-133 mimic resulted in significant down-regulation of *I*. *scapularis henna* transcript levels, suggesting conserved nature of this miRNA binding to its target gene in Locusts and ticks. In addition, we noted that *A*. *phagocytophilum* has no impact on regulation of *henna* gene expression. These observations suggest that *A*. *phagocytophilum* infection results in specific modulation of arthropod miR-133-mediated *isoatp4056* gene expression important for this bacterial survival in ticks and for its transmission to the vertebrate host.

Increased expression of miR-133 expression in larval and nymphal ticks in comparison to adult ticks suggests an important role for miR-133-mediated signaling in early developmental stages of these ticks. We also noted that in addition to miR-133, other miRNAs were down-regulated in *A*. *phagocytophilum*-infected ticks in comparison to the levels noted in uninfected ticks. The down-regulation of other miRNAs in *A*. *phagocytophilum*-infected ticks suggests their involvement in controlling gene expression of other target genes that could be essential in tick-rickettsial pathogen associations. Future studies will unravel the roles for these individual miRNAs and their target genes in tick-*A*. *phagocytophilum* interactions.

The silencing of gene expression by miRNA binding could be due to mRNA degradation or translational repression of target genes [37, 40]. Our studies with pmirGLO constructs revealed that miR-133-mediated regulation of *isoatp4056* is possibly due to RNA degradation of this target gene. The down-regulation of miR-133 and upregulation of *isoatp4056* transcripts during transmission of *A*. *phagocytophilum* from vector to vertebrate host, suggests that down-regulation of miRNA is critical for successful passage of this bacteria from vector to the vertebrate host. The observation of less bacterial burden in mice infested with pre-mir-133-treated ticks in comparison to the bacterial loads noted in mice infested with mock-treated ticks suggests that IsOATP4056 is critical for *A*. *phagocytophilum* transmission from vector to the vertebrate host.

The pre-mir-133 is the precursor form that has to be processed to form mature miR-133 for binding to target sites. Some of the experiments in this current study were performed with pre-mir-133. Our studies showed that treatment of ticks or tick cells with pre-mir-133 showed significant silencing of *isoatp4056* expression. This observation was also noted with the use of mature miR-133 mimic. The synthesis of miRNA from pri-miRNA to mature form involves participation of several molecules including RNA polymerase II, ribonuclease III, dicer, Ago2 and other RISC-associated proteins [37, 40, 45, 47]. Evidence for the presence of these proteins in *I*. *scapularis* [66] suggests that exogenous pre-mir-133 could be processed to mature miR-133 in ticks and tick cells. The observation of no toxic effects of pre-mir-133 to tick cells *in vitro* or to ticks during feeding suggests that miR-133 does not affect tick fitness but rather affects regulation of *isoatp4056* that is important for *A*. *phagocytophilum* survival in these cells.

**Fig 9:**
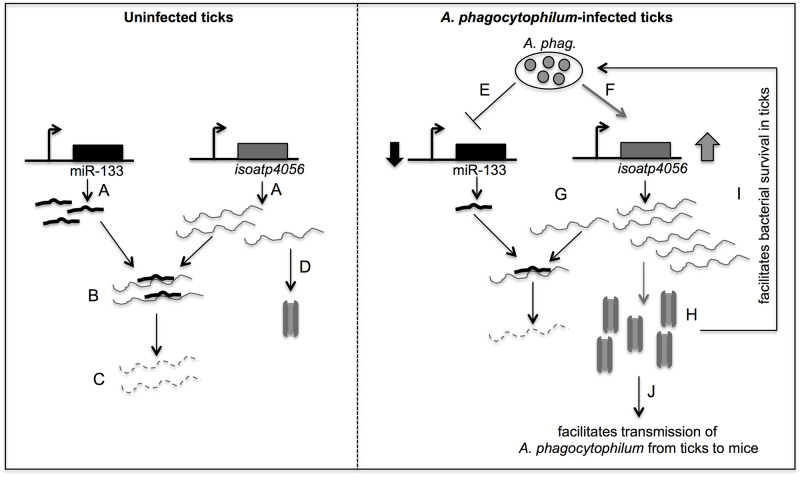
Model showing *A*. *phagocytophilum*-mediated regulation of miR-133 to enable its survival in the vector host and its transmission to vertebrate host. In the uninfected state, both miR-133 and *isoatp4056* are expressed at endogenous level (A). The level of *isoatp4056* expression is maintained by the binding of miR-133 to the target region (B) leading to mRNA degradation (C). The endogenous levels of Isoatp4056 are maintained in the cells by translation of any remaining mRNA that is not bound by miR-133 (D). In the infected state, *A*. *phagocytophilum* down-regulates miR-133 (E) and up-regulates *isoatp4056* transcript levels (F) that results in less production of miR-133 and enhanced production of *isoatp4056* mRNA (G). The availability of reduced miR-133 to bind *isoatp4056* mRNA subsequently leads to increased production of IsOATP4056 protein (H) that is critical for both bacterial survival in the vector host (I) and for its transmission to the vertebrate host (J). The picture is not drawn to scale.

Based on the current findings and our previous report [26], we propose a model (Fig 9) that suggests that in uninfected ticks the binding of miR-133 to *isoatp4056* transcripts maintains the endogenous levels of IsOATP4056 protein. The production of endogenous level of tick IsOATP4056 protein could be sufficient in normal conditions. However, upon *A*. *phagocytophilum* infection, the down-regulation of miR-133 enhance *isoatp4056* transcript levels. The availability of increased *isoatp4056* transcripts and decreased miR-133 will enable production of more of IsOATP4056 protein that supports *A*. *phagocytophilum* survival in its vector and for its transmission to the vertebrate host (Fig 9). We hypothesize that the down-regulation of miR-133 may be due to binding of *A*. *phagocytophilum* effector proteins to miR-133 promoter that could lead to inhibition of promoter activation. Alternatively, *A*. *phagocytophilum* infection may induce other tick transcriptional repressors to bind miR-133 promoter and inhibit promoter activation. With any of these hypotheses, the findings from this study provides an interesting model to understand how vector-borne pathogens modulate gene regulation in their vectors for their survival and transmission to the vertebrate host. To our knowledge, this is the first report that shows significance of miRNA down-regulation in pathogen transmission from ticks to vertebrate host.

In conclusion, we provide substantial novel evidence that rickettsial pathogen *A*. *phagocytophilum* infection results in down-regulation of arthropod miR-133 expression that enhances *isoatp4056* transcript levels required for this bacterial survival in ticks and for its transmission from vector to the vertebrate host. Understanding how pathogens manipulate vector-signaling repertoire for their benefit would lead to the development of strategies to block their transmission from vector to the vertebrate host.

## Materials and methods

### Ethics statement

All animal work in this study was carried out in strict accordance with the recommendations in the Guide for the Care and Use of Laboratory Animals of the National Institute of Health. The Old Dominion University Animal husbandry facilities and University Institutional Animal Care and Use Committee (IACUC) approved protocol (permit number 16–017 and 19–009) was followed in all mice experiments. Acepromazine tranquilizer was administered to the mice prior to handling to minimize anxiety and/or discomfort and all efforts were made to minimize suffering.

### Ticks, cells and bacterial isolates

All developmental stages of *I*. *scapularis* ticks (larvae, nymphs, adult male and female) used in this study were either obtained from BEI Resources, NIAID, NIH or from Connecticut Agricultural Experiment Station (New Haven, CT). We generated infected nymphs by feeding uninfected larvae on *A*. *phagocytophilum*-infected mice. These fed larvae were then allowed to molt to nymphs. Tick rearing was conducted in an incubator at 23 ± 2°C with 95% relative humidity and a 14/10 hour light/dark photoperiod regiment. The molted *A*. *phagocytophilum* unfed nymphs were used in expression analysis and transmission experiments. ISE6 tick cell line (ATCC) used in this study was maintained as described [23, 26, 28]. The *A*. *phagocytophilum* NCH-1 isolate (obtained from BEI resources) was used in the *in vivo* tick & mice experiments and *in vitro* cell culture studies. *Anaplasma phagocytophilum* isolate was maintained in HL-60 (ATCC) cells as described [23, 26]. The *Escherichia coli* JM109 strain was used as a cloning host for generating pmirGLO constructs used in luciferase assays. The *E*. *coli* DH5α strain was used for cloning full-length *isoatp4056* gene.

### Mice and tick feeding

C3H/HeN mice (CharlesRiver Laboratories, USA) were used for transmission experiments. Unfed uninfected or *A*. *phagocytophilum*-infected nymphal ticks were fed on these mice. *Anaplasma phagocytophilum* infection was maintained in B6.129S7-Rag1tm1Mom/J mice (Jackson Laboratories, USA). To generate uninfected or *A*. *phagocytophilum*–infected unfed nymphs, larvae were fed on either uninfected or *A*. *phagocytophilum*–infected mice and allowed to molt as described [23, 26]. To analyze expression of miR-133 and *isoatp4056* in ticks during transmission, uninfected or *A*. *phagocytophilum*-infected unfed nymphs were fed on naïve mice. Repleted fed ticks were collected and processed for miRNA or total RNA isolations followed by Quantitative real time PCR (QRT-PCR) analysis. To analyze the effect of pre-mir-133 treatment on bacterial transmission from ticks to murine host, pre-mir-133- or mock-treated *A*. *phagocytophilum*-infected unfed nymphs were fed on naïve mice. Murine blood and spleen was collected after day 10 post-tick placement on mice and analyzed for bacterial burden.

### MicroRNA prediction from tick genome

The genomic sequences of *I*. *scapularis* were downloaded from NCBI bio-project (https://www.ncbi.nlm.nih.gov/bioproject/531218) and from VectorBase database. All previous reported mature animal miRNA and precursor sequences were obtained from miRBase (version 21 released in June 2014; available at http://www.mirbase.org/). This miRNA dataset was used as query sequences for BLAST based homology search against tick genome and was carried out in BioEdit version 7.2.5 with an E value ≤0.01 and default value for the other parameters as described [46]. All the selected pre-miRNA sequences were then submitted to Mfold online software (http://mfold.rna.albany.edu/?q=mfold/RNA-Folding-Form) to predict secondary structure (hairpin) and calculate free energy (ΔG). The minimal folding free energy index (MFEI) was calculated to avoid designating other RNAs as miRNAs. Identification of *I*. *scapularis* miR-133 targets was accomplished using MiRanda version 3.3a (http://www.microrna.org/) and RNAhybrid version 2.1.2 (http://bibiserv.techfak.unibielefeld.de/rnahybrid/) with the parameters described in the previous study [46].

### Phylogenetic analysis

Phylogenetic analyses were performed for *I*. *scapularis* miR-133 families with various organisms. The phylogenetic tree was drawn using the Neighborhood Joining (NJ) method with 1000 bootstrap replicates using MEGA version 7.0 software to demonstrate evolutionary relationships among various species.

### Isolation of microRNA, Total RNA, DNA isolation and QRT-PCR data analysis

MicroRNA extractions from *I*. *scapularis* larvae, nymphs, adult male, adult female and ISE6 tick cells *in vitro* were performed using mirPremier microRNA isolation kit (Sigma, USA) following the manufacturer’s instructions. The cDNA was generated from microRNA using the MystiCq microRNA cDNA Synthesis Mix kit (Sigma, USA) and used as template for tick microRNA expression analysis. Total RNA from tick unfed/fed nymphs and ISE6 tick cells (2 x 10^5^) were extracted using Aurum Total RNA Mini kit (BioRad, USA) following manufacturer instructions. The cDNA was generated from total RNA using iScript cDNA synthesis kit (BioRad, USA) and used as template for the amplification of *isoatp4056*, *henna* and housekeeping genes with oligonucleotides as mentioned in S1 Table. All oligonucleotides used in this study are mentioned in S1 Table. QRT-PCR was performed using iQ-SYBR Green Supermix and CFX96 touch System (BioRad, USA). As an internal control and to normalize the amount of template, tick beta actin amplicons were quantified using published primers [23, 26]. *A*. *phagocytophilum* burden was quantified in DNA samples using published primers [23, 26] or with the primers listed in S1 Table. DNA from ticks or tick cells was extracted using Qiagen DNeasy kit (Qiagen, USA). The standard curves were prepared using 10-fold serial dilutions starting from 1 to 0.000001 ng/μl of known quantities of respective gene fragments.

### Full-length *isoatp4056* gene isolation

The nucleotide sequence for tick *isoatp4056* gene from the genomic region (GenBank acc. no. DS922985) and partial transcript sequence (GenBank acc. no. XM_002414056.1) was downloaded from NCBI and was used to predict the possible 3’ UTR region using *Drosophila melanogaster oatp* sequence as a reference gene. Using a set of oligonucleotides (as mentioned in S1 Table), *isoatp4056* complete CDS along with 3’UTR region was amplified from cDNA prepared from adult female tick RNA. The PCR amplified tick *isoatp4056* full-length gene fragment was cloned into pGEM-T-easy vector (Promega, USA) for sequencing and storage. Nucleotide sequencing was performed at Eurofins Genomics (Eurofins, USA) and analyzed using DNASTAR Lasergene 10 (DNASTAR, USA).

### Pre-microRNA synthesis and tick microinjections

The precursor sequence of miR-133 was downloaded from the miRbase and T7 promoter sequences was added at 5’ end. The entire oligonucleotide was synthesized and was used as a DNA template for *in vitro* microRNA synthesis. MicroRNA synthesis were performed using MEGAscript RNAi Kit (Ambion Inc. USA) following manufacturer instructions. Microinjections (~4.2 nl/tick) with pre-mir-133 (100 ng/μl), miR-133 mimic (12.5 nmoles/μl) or miR-133 inhibitor (25 nmoles/μl) or mock solution were performed as described [23, 26]. After microinjection, ticks were incubated for 48 hours in a desiccator (for recovery) housed in an incubator set at 23 ± 2°C with 95% relative humidity and a 14/10 hour light/dark photoperiod regimen. Microinjected ticks were later fed on uninfected mice. Engorged ticks were collected after repletion and RNA or DNA extractions were performed. QRT-PCR was performed to determine microRNA-based target gene silencing efficiency, gene expression, and bacterial burden.

### Salivary glands and gut isolation

Individual fed uninfected or *A*. *phagocytophilum*-infected nymphal tick salivary glands or whole guts were dissected in sterile 1x phosphate buffer saline. One set of these samples was processed for homogenization in lysis buffer (Aurum Total RNA kit, Bio-Rad) for RNA extractions following manufacturer’s recommendation. The other set was pooled for isolation of miRNA using PureLink miRNA isolation kit (ThermoFisher Scientific). The extracted RNA or miRNA was processed for cDNA synthesis using iSCRIPT cDNA synthesis kit (BioRad) or qScript microRNA cDNA Synthesis Kit (QuantaBio, VWR). QRT-PCR was performed with these cDNAs to quantify miR-133 or *isoatp4056* expression.

### Tick cell line experiment with pre-microRNA

For microRNA-based silencing of *isoatp4056* expression in ISE6 tick cells, Lipofectamine 2000 transfection reagent (ThermoFisher Scientific/Invitrogen) was used. Briefly, 2 × 10^5^ tick cells were seeded in L-15B300 medium on to 12 well plates and incubated for 24 hours. After 24 h, 700 ng of pre-mir-133 mixed with Lipofectamine reagent was added to the cells. After 6 hours of addition of pre-mir-133 and Lipofectamine, 2x FBS L15-B300 medium was added and plates were further incubated for additional 16 h. Cell-free *A*. *phagocytophilum* (isolated from infected HL-60 cells) was added after 24 h post-transfection and tick cells were incubated further for 24 h and processed for RNA or DNA extractions to analyze the silencing efficiency and bacterial burden, respectively.

### Tick cell live/dead assay

The effects of miR-133 on tick cells were analyzed by using LIVE/DEAD Cell Imaging kit (Molecular Probes, Life technologies) following manufacturer instructions. Briefly, 2 × 10^5^ tick cells were seeded in L-15B300 medium on to 12 well plates and incubated for 24 hours. After 24h, 700 ng of pre-mir-133 mixed with Lipofectamine reagent was added to the cells. After 6 hours, 2x FBS L15-B300 medium was added and plates were further incubated for additional 24 and 48 h. Cells were processed for live/dead staining and imaged at GFP channel and Texas Red channel with EVOS imaging system (Invitrogen/ThermoScientific Inc.). Different images were captured and percentage of live cells was calculated based on the number of dead cells/total cells per image. At least a minimum of 5 images was considered to calculate percentage of live cells for each sample. Image J (NIH) software was used to merge images taken in GFP and Texas Red channel from EVOS imaging system to generate overlay images shown in figures.

### Dual-Luciferase assays

The Dual-Luciferase (pmirGLO, Promega, USA) constructs were transfected into ISE6 tick cells to validate the miR-133 binding on the target gene. The pmirGLO Dual-Luciferase miRNA target expression vector was cloned with match and mismatch target site for miR-133, using PmeI and XbaI double digestion. The positive clones were confirmed with NotI based digestion following manufacturer instructions and sequenced. Constructs carrying either an exact match to the 22 bp miR-133 target sequence (from *isoatp4056*) or a mismatched version of that target site were mixed with 500 ng of pre-mir-133 and transfected into ISE6 tick cells using Lipofectamine reagent. After 24 hours incubation, cells were harvested and estimated for luciferase activity using luminometer (M200 Infite PRO, Tecan, USA). Luciferase activity (Firefly luciferase activity/Renilla luciferase activity) for experimental groups transfected with different constructs was normalized to that of the luciferase activity from cells transfected with empty pmirGLO vector control (no-insert control). For each transfection, luciferase activity was averaged from six replicates. Total RNA was also extracted from tick cells and used as template to measure Firefly and Renilla luciferase transcripts.

### Statistics

All the data set were statistically analyzed using Microsoft Excel 2016 and GraphPad Prism 6 software. The non-paired Student t-test was considered to compare the experiment data sets with two variables and ANOVA was considered for comparing data obtained from more than two variables. P values of <0.05 were considered as significant for the data analyzed with both analysis. The P values obtained in the data analyses were shown in appropriate figure and panels.

### Accession numbers

Isc-miR-133-1 (miRBase acc. no. MI0012266), isc-miR-133-2 (GenBank acc. no. DS613658), *Rhipicephalus* microplus miR-133-1 (GenBank acc. no. LYUQ01278569), *R*. *microplus* miR-133-2 (GenBank acc. no. LYUQ01042747), *Anopheles gambiae* miR-133 (miRBase acc. no. MI0001606), *Aedes aegypti* miR-133 (miRBase acc. no. MI0013482), *Culex quinquefasciatus* miR-133 (miRBase acc. no. MI0013625), *Homo sapiens* miR-133a-1 (miRBase acc. no. MI0000450), *H*. *sapiens* miR-133a-2 (miRBase acc. no. MI0000451), *Mus musculus* miR-133a-1 (miRBase acc. no. MI0000159), *M*. *musculus* miR-133a-2 (miRBase acc. no. MI0000820), *I*. *scapularis isoatp4056* (GenBank acc. no. XM_002414056.1), *I*. *scapularis* U6 splicesomal RNA (GenBank acc. no. DS675898), *I*. *scapularis henna* (GenBank acc. no. XM_002406604.1) *I*. *scapularis* 5.8S rRNA (GenBank acc. no. GU353340), *I*. *scapularis isoatp4056* (GenBank acc. no. MT152669).

## Supporting information

S1 Fig Secondary structure of *I*. *scapularis* miRNAs. Mfold online tool was used to draw the stem-loop structure of the pre-miRNA and calculate free energy (ΔG in kcal/mol). Mature miRNA sequences in the stem-loop are highlighted and ΔG values for each pre-miRNA are indicated.
(TIFF)

S2 Fig *A*. *phagocytophilum* does not alter miR-9b and miR-10 levels in ticks. QRT-PCR analysis showing levels of miR-9b (A) and miR-10 (B) in unfed uninfected (UI) or *A*. *phagocytophilum*-infected (I) nymphal ticks. The levels of miRNA were normalized to tick 5.8S rRNA. Open circles indicate data from uninfected ticks and closed circles indicate data from *A*. *phagocytophilum*-infected ticks. Each circle represents sample generated from pool of three nymphs. QRT-PCR analysis showing levels of miR-133 (C) and bacterial loads (D) in *A*. *phagocytophilum*-infected fed larvae and unfed nymphs. Statistical analysis was performed using Student’s t test and P value is shown.
(TIFF)

S3 Fig Ticks cells have no detectable levels of miR-133. Melt peak from QRT-PCR assay showing enhanced levels of miR-133 in unfed uninfected nymphs in comparison to levels noted in ISE6 tick cells. No detectable levels of miR-133 were noted in ISE6 tick cells. NTC indicates melt peak from no template control reaction.
(TIFF)

S4 Fig In vitro Synthesis of precursor microRNA (pre-mir-133). (A) A 104bp of DNA oligonucleotide with T7 promoter sequence at 5’ end is shown. (B) Agarose gel image showing DNase and RNase (single strand specific) digested product to confirm pre-mir-133 dsRNA formation after *in vitro* transcription using MEGAscript RNAi kit.
(TIFF)

S5 Fig QRT-PCR analysis showing levels of *isoatp4056* in pre-mir-133 treated ticks. A) QRT-PCR analysis showing level of *isoatp4056* in pre-mir-133-treated or mock-treated unfed nymphs at 48 h post treatment is shown. Open circles indicate mock-treated ticks and closed circle indicates pre-mir-133-treated ticks. B) QRT-PCR analysis showing levels of *henna* transcripts in uninfected (UI) and *A*. *phagocytophilum*-infected (I) unfed nymphal ticks is shown. Open circles indicate uninfected ticks and closed circles indicate *A*. *phagocytophilum*-infected ticks. Each circle represents one individual tick. Expression levels of *isoatp4056* or *henna* were normalized to tick beta-actin levels. Statistical analysis was performed using non-paired student’s t-test and P value is shown.
(TIFF)

S6 Fig pre-mir-133 is not toxic to tick cells at 24 h post-treatment time point. A) Fluorescent microscopic images showing live (green) and dead (red) tick cells. Tick cells were treated with pre-mir-133 for 24 h and processed for staining using Live/Dead staining kit followed by imaging using EVOS fluorescent microscope. Scale bar indicates 200 μm. B) Quantification of number of live cells in mock-trerated or pre-mir-133 treated tick cells at 24 and 48 h post-treatment is shown.
(TIFF)

S7 Fig Expression of miR-133 and *isoatp4056* in *A*. *phagocytophilum*-infected tick guts during transmission. QRT-PCR analysis showing level of miR-133 (A) and *isoatp4056* (B) in guts isolated from uninfected or *A*. *phagocytophilum*-infected nymphal ticks after feeding on naïve mice. Open circles indicate samples generated from uninfected ticks and closed circle indicate samples generated from *A*. *phagocytophilum*-infected ticks.
(TIFF)

S1 Table Oligonucleotides used in this study. Table shows oligonucleotides used in this study. The sequence orientation is from 5’ to 3’ end. The purpose for which oligonucleotides used is mentioned.
(PDF)

S2 Table Identification of miRNAs from *I*. *scapularis* genome. New and previously identified *I*. *scapularis* miRNA analyzed in this study are shown. Mature sequence is provided. In addition, *I*. *scapularis* genome GenBank accession numbers are shown. GC percentage and pre-mature sequence and size are also shown.
(PDF)

S3 Table ANOVA analysis for Figs 5, 7A and 7B. Table shows P value from one-way ANOVA analysis for all the data shown in Figs 5, 7A and 7B.
(PDF)
