# Supplementary figures and images for: Repression of tick microRNA-133 induces organic anion transporting polypeptide expression critical for Anaplasma phagocytophilum survival in the vector and transmission to the vertebrate host
Source: PLoS Genet. 2020 Jul 2;16(7):e1008856. doi: 10.1371/journal.pgen.1008856 (PMC7331985; doi:10.1371/journal.pgen.1008856)

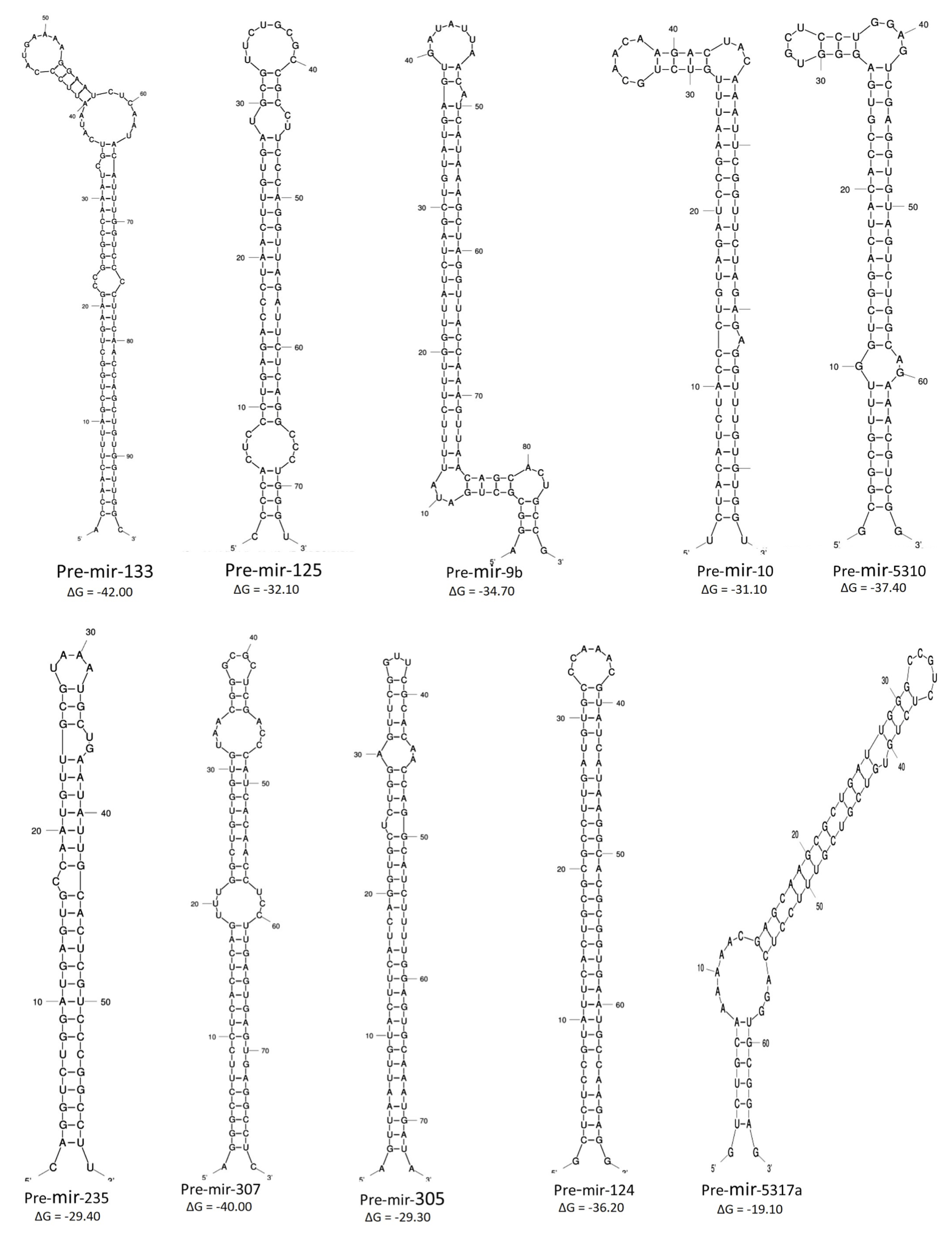

Supplement: S1 Fig — Mfold online tool was used to draw the stem-loop structure of the pre-miRNA and calculate free energy (ΔG in kcal/mol). Mature miRNA sequences in the stem-loop are highlighted and ΔG values for each pre-miRNA are indicated. (TIFF) [file pgen.1008856.s001.tiff]

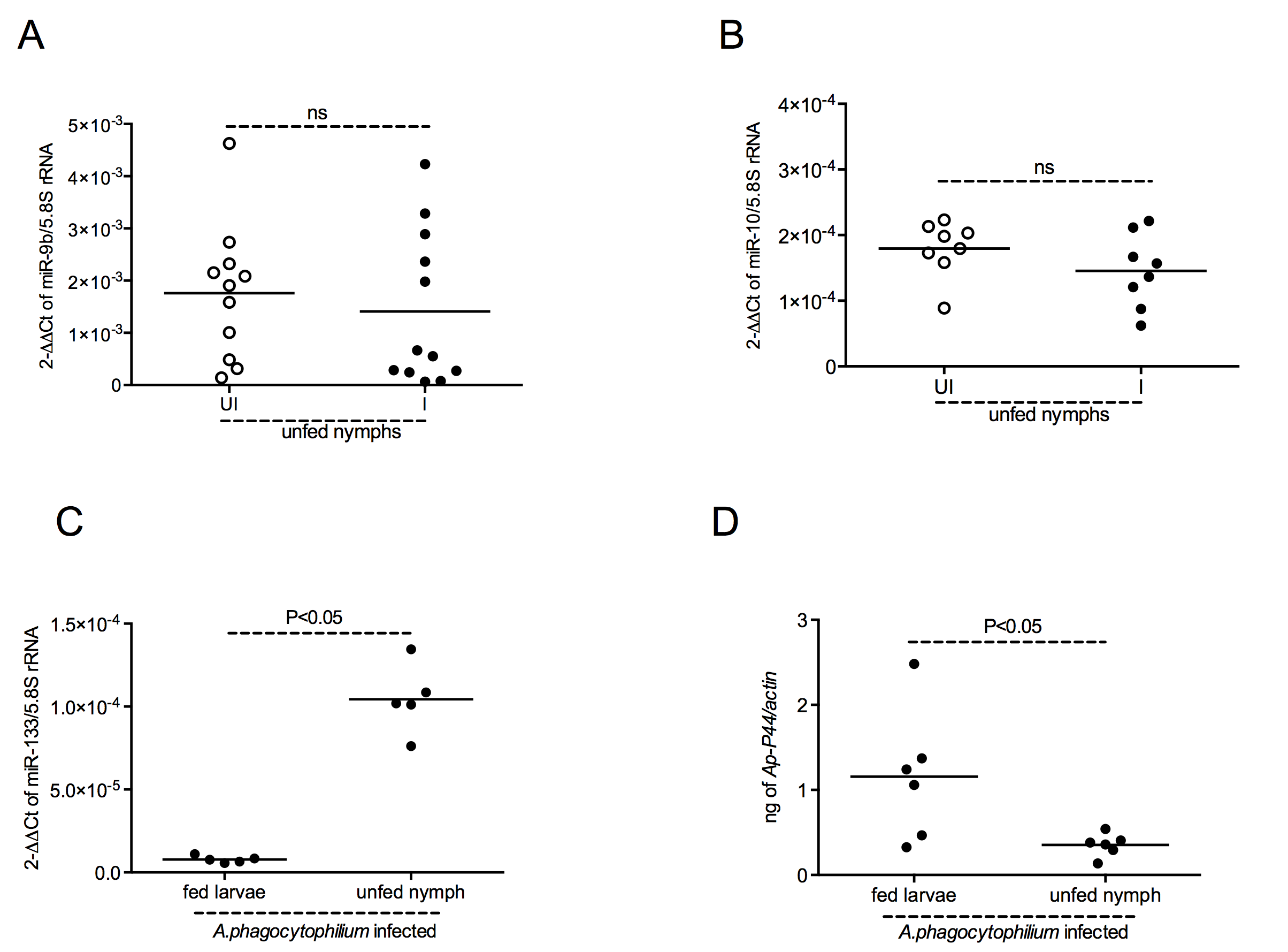

Supplement: S2 Fig — QRT-PCR analysis showing levels of miR-9b (A) and miR-10 (B) in unfed uninfected (UI) or A. phagocytophilum-infected (I) nymphal ticks. The levels of miRNA were normalized to tick 5.8S rRNA. Open circles indicate data from uninfected ticks and closed circles indicate data from A. phagocytophilum-infected ticks. Each circle represents sample generated from pool of three nymphs. QRT-PCR analysis showing levels of miR-133 (C) and bacterial loads (D) in A. phagocytophilum-infected fed larvae and unfed nymphs. Statistical analysis was performed using Student’s t test and P value is shown. (TIFF) [file pgen.1008856.s002.tiff]

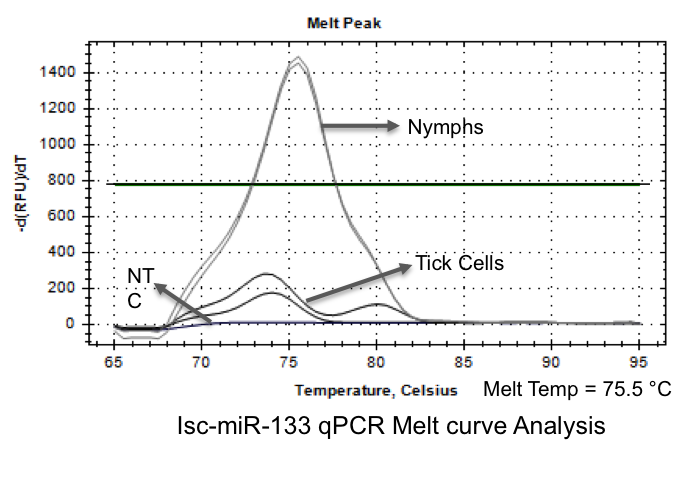

Supplement: S3 Fig — Melt peak from QRT-PCR assay showing enhanced levels of miR-133 in unfed uninfected nymphs in comparison to levels noted in ISE6 tick cells. No detectable levels of miR-133 were noted in ISE6 tick cells. NTC indicates melt peak from no template control reaction. (TIFF) [file pgen.1008856.s003.tiff]

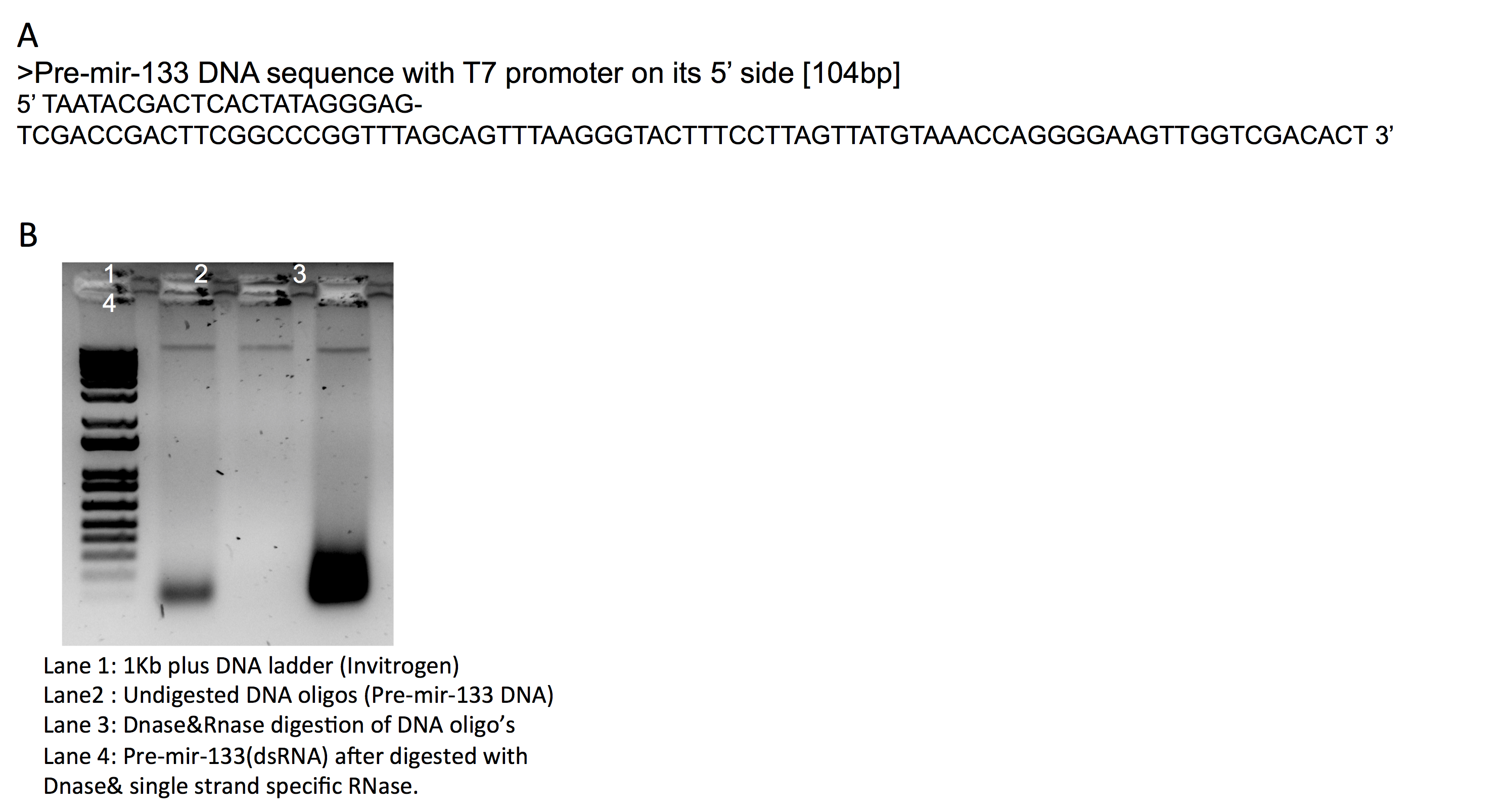

Supplement: S4 Fig — (A) A 104bp of DNA oligonucleotide with T7 promoter sequence at 5’ end is shown. (B) Agarose gel image showing DNase and RNase (single strand specific) digested product to confirm pre-mir-133 dsRNA formation after in vitro transcription using MEGAscript RNAi kit. (TIFF) [file pgen.1008856.s004.tiff]

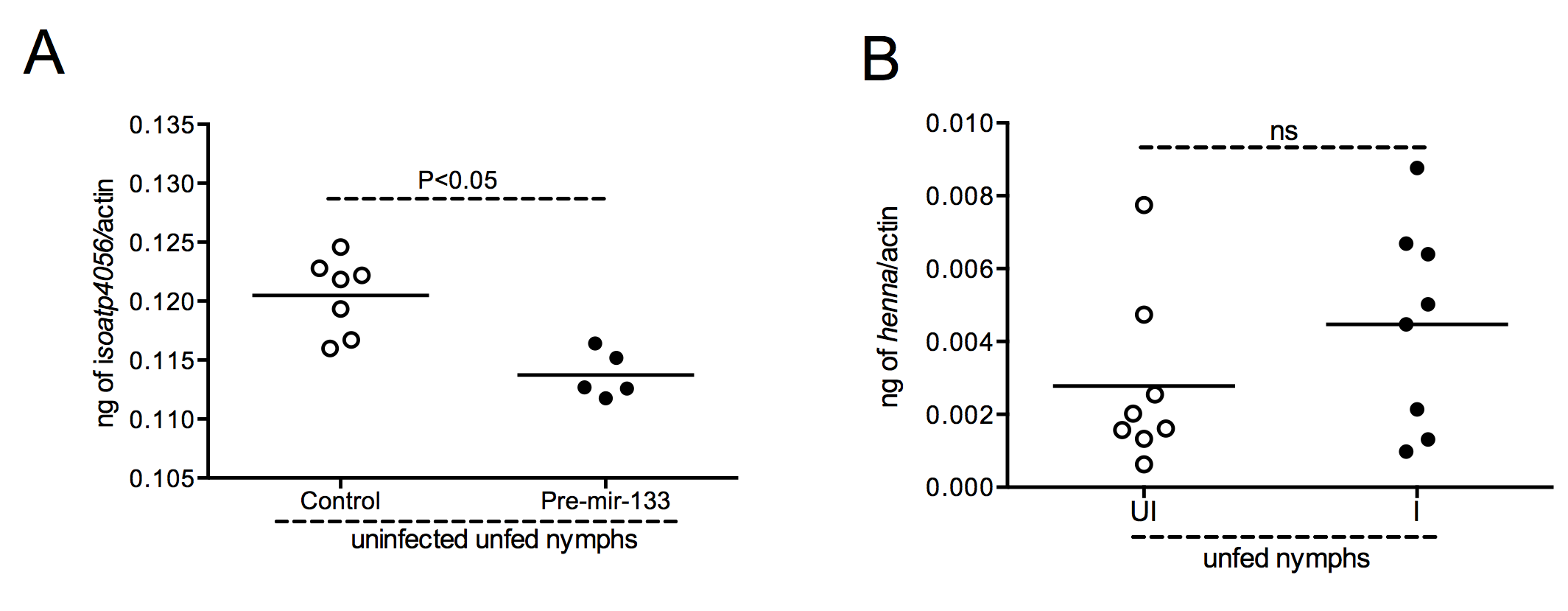

Supplement: S5 Fig — A) QRT-PCR analysis showing level of isoatp4056 in pre-mir-133-treated or mock-treated unfed nymphs at 48 h post treatment is shown. Open circles indicate mock-treated ticks and closed circle indicates pre-mir-133-treated ticks. B) QRT-PCR analysis showing levels of henna transcripts in uninfected (UI) and A. phagocytophilum-infected (I) unfed nymphal ticks is shown. Open circles indicate uninfected ticks and closed circles indicate A. phagocytophilum-infected ticks. Each circle represents one individual tick. Expression levels of isoatp4056 or henna were normalized to tick beta-actin levels. Statistical analysis was performed using non-paired student’s t-test and P value is shown. (TIFF) [file pgen.1008856.s005.tiff]

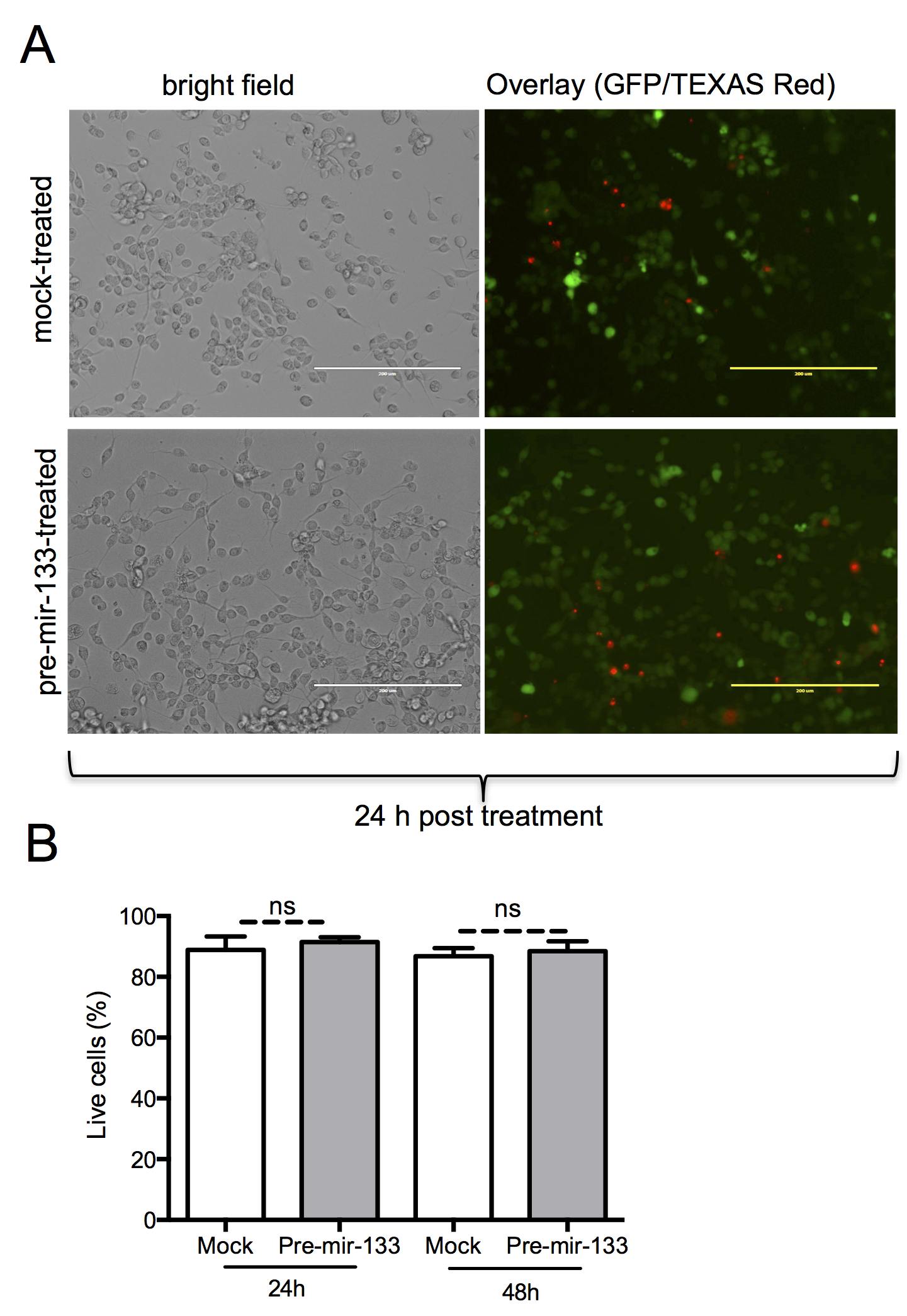

Supplement: S6 Fig — A) Fluorescent microscopic images showing live (green) and dead (red) tick cells. Tick cells were treated with pre-mir-133 for 24 h and processed for staining using Live/Dead staining kit followed by imaging using EVOS fluorescent microscope. Scale bar indicates 200 μm. B) Quantification of number of live cells in mock-trerated or pre-mir-133 treated tick cells at 24 and 48 h post-treatment is shown. (TIFF) [file pgen.1008856.s006.tiff]

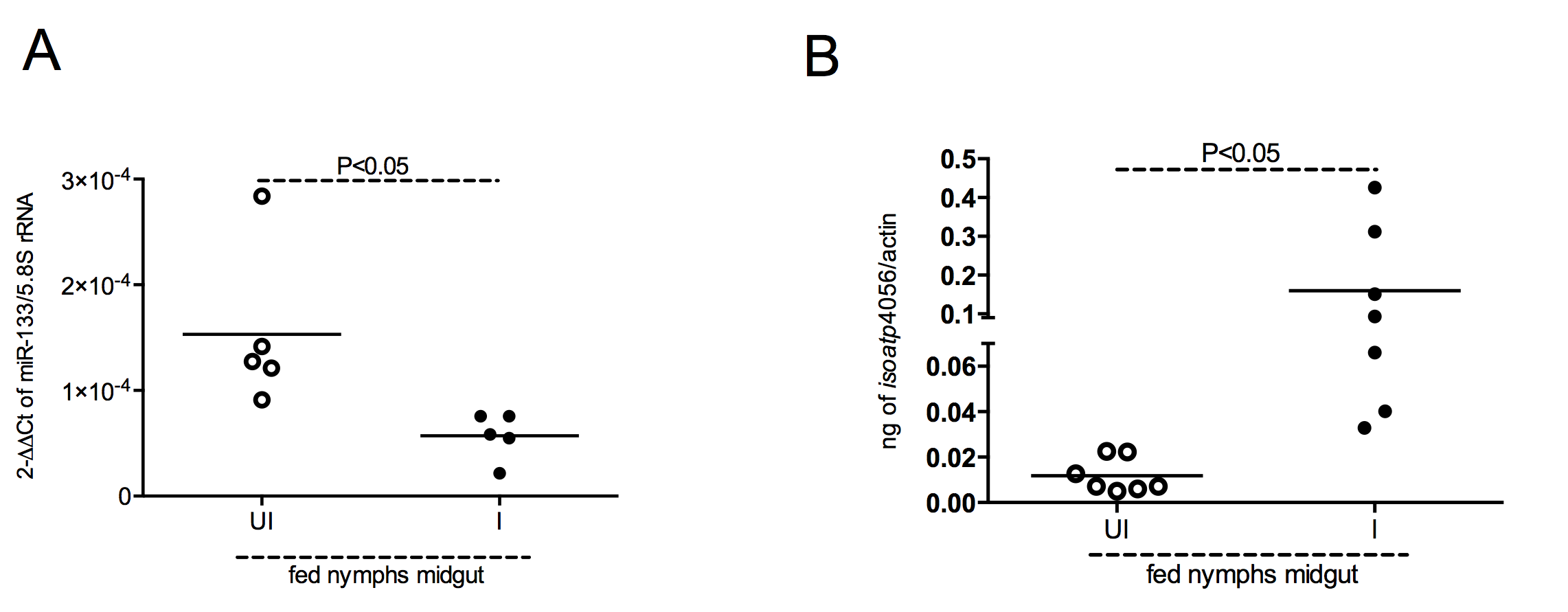

Supplement: S7 Fig — QRT-PCR analysis showing level of miR-133 (A) and isoatp4056 (B) in guts isolated from uninfected or A. phagocytophilum-infected nymphal ticks after feeding on naïve mice. Open circles indicate samples generated from uninfected ticks and closed circle indicate samples generated from A. phagocytophilum-infected ticks. (TIFF) [file pgen.1008856.s007.tiff]
