# Supplementary material for: Repression of tick microRNA-133 induces organic anion transporting polypeptide expression critical for Anaplasma phagocytophilum survival in the vector and transmission to the vertebrate host
Source: PLoS Genet. 2020 Jul 2;16(7):e1008856. doi: 10.1371/journal.pgen.1008856 (PMC7331985; doi:10.1371/journal.pgen.1008856)
Supplement: S1 Table — Table shows oligonucleotides used in this study. The sequence orientation is from 5’ to 3’ end. The purpose for which oligonucleotides used is mentioned. (PDF) [file pgen.1008856.s008.pdf]

S1 Table. Oligonucleotides used in this study

**Oligo Sequence 5' to 3'**	**Purpose**
GGTATCGTGCTCGACTC	*Ixodes scapularis actin* gene, qPCR
CAGGGCGACGTAGCAG	
CCCTAGGCACCAGGGTGTGA	Mice actin gene, qPCR
GGGGTGTTGAAGGTCTCAAACA	
CCAGCGTTTAGCAAGATAAGAG	*Anaplasma phagocytophilum -p44* gene
GCCCAGTAACAACATCATAAG	
GCGGTTCATAAAGGAGTGGA	*Anaplasma phagocytophilum -dnaA* gene
AAACTTCCTGCGCCTCTGTA	
TAAACAATTAAAAGCTTTCTT	Tick 16s rRNA (mt), qPCR
AATCGCTAAAAACGGAACTTA	
CCGTCACGAAAACGCCTTCA	*I. scapularis isoatp4056* qPCR
GCTTCCACACGTCCACCTTCT	
TGGGATCCATGGCGTCCAGCTGCCT	*I. scapularis isoatp4056* gene (Complete Coding part), Sequencing
TGGTCGACGAGTATGTCTCAACACCTCGCA	
GGGTGTTCCACTGCACTCAGTA	*I. scapularis henna* gene (*phenylalanine hydroxylase*) qPCR
GCACGTAGCCAATTTCTCAATC	
GCACAAGCGGGAACTCAAGGA	*isoatp4056* 3' UTR region, sequencing
GTCATCCTGCAACTACCAGAGAGA	
GTCTGGCCTTTCACTACTCCTA	Renilla luciferase (pmirGLO vector), qPCR
GAGCATGGTCTCGACGAAGA	
GCCAAACGCTTCCACCTACCA	Firefly luciferase (pmirGLO vector), qPCR
GGTTCACACCCAGTGTCTTACC	
CCAGTGCAAATGACCAACTTTAG	isc-miR-133 gene, sequencing
CTTCACGAACGCACAGAAGGA	
CACCTTATCAGCGAGTCATCAA	isc-miR-124 gene, sequencing
CCGGAACACATCTTCCTGATTG	
GCTTCGGCAGTACATATACTAA	U6 spliceosomal RNA, sequencing
CTTCACGATATTGCGTGTCAT	
TCTAAGCGGTGGATCACTCGGT	5.8s ribosomal gene, sequencing
GACCCTCACACAGACGAAGCCA	
TCGTTAGTCCCCTTCAACCAG	isc-miR-133 qPCR
TCGTTGGTTATCTAGCTGTATGA	isc-miR-9b qPCR
TAATACACACGGTGAATGCCAA	isc-miR-124 qPCR
CACAACCTCCTTGAGTGAGTGA	Isc-miR-307 qPCR
TATTGTACTCGTCCCGGCCTT	Isc-miR-235 qPCR
GGACGATACAGAGAAGATTAGCA	U6 RNA qPCR
GCGTACCCTGTAGATCCGAA	isc-miR-10 qPCR
TCCCTGAGACCCTAACTTGTGA	isc-miR-125 qPCR
GCGAGGTAGTAGGTTGTATAGT	isc-let7 qPCR
GTGTAGTCTGGCAGAAACGTC	isc-miR-5310 qPCR
CGTGTACTTCATCAGGTGCTCT	isc-miR-305 qPCR

**Oligo Sequence 5' to 3'**	**Purpose**
TCTTGTCTCTGTGTCGTCGTT	isc-miR-5317a
MIRUP-MystiCq® Universal PCR Primer (Sigma, USA)	MicroRNA qPCR Universal Reverse
AAAC TA GCGGCCGC TAGT CAAGCTGGTTGGGCTGGGCCAG T	isc-miR-133-targetSite (*isoatp4056* -22bp) Sense and Anti sense oligo's
CTAGA CTGGCCCAGCCCAACCAGCTTG ACTA GCGGCCGC TA GTTT	
AAAC TA GCGGCCGC TAGT CAAGCTGGTTGGGCTGAACAG T	isc-miR-133 mismatch site Sense and Anti sense oligo's
CTAGA CTGTTCAGCCCAACCAGCTTG ACTA GCGGCCGC TA GTTT	
TAATACGACTCACTATAGGGAG - TCGACCGACTTCGGCCCGGTTTAGC AGTTTAAGGGTACTTTCCTTAGTTATGT AAACCAGGGGAAGTTGGTCGACACT	pre-mir-133 precursor sequence with T7 promotor at 3'UTR (DNA template)
