# Supplementary material for: Repression of tick microRNA-133 induces organic anion transporting polypeptide expression critical for Anaplasma phagocytophilum survival in the vector and transmission to the vertebrate host
Source: PLoS Genet. 2020 Jul 2;16(7):e1008856. doi: 10.1371/journal.pgen.1008856 (PMC7331985; doi:10.1371/journal.pgen.1008856)
Supplement: S2 Table — New and previously identified I. scapularis miRNA analyzed in this study are shown. Mature sequence is provided. In addition, I. scapularis genome GenBank accession numbers are shown. GC percentage and pre-mature sequence and size are also shown. (PDF) [file pgen.1008856.s009.pdf]

S2 Table. Identification of miRNAs from *I. scapularis* genome.

**miRNA**	**mature Sequence**	**miRBase Accession Number**	***Ixodes* genome ID**	**Blast Hit info**	**strand**	**Pre-Mature Seq**	**MFE**	**bp**	**G+C %**	**MFE I**
rmi-miR-9b	UCUUUGGU UAUCUAGC UGUAUGA	New Finding	DS885551	436513 to 436535	plus	AGGCGCTGATATTTTCTTTGGTTA TCTAGCTGTATGAGTGATATTAAC ATCATAAAGCTAGGTTACCAAAG TTAACAGCACTGCCG	-34.70	86	38.4	-1.05
hlo-miR-307	UCACAACCU CCUUGAGU GAG	New Finding	DS911299	1700331 to 1700311	minus	AGGGCCTTCCTCACTCAGTTTGGC TGTGGTGTAACGGGCGCTCGACCC ATCACAACCTCCTTGAGTGAGTGA GGCCTC	-40.00	78	59.0	-0.87
prd-miR-235	UAUUGCAC UCGUCCCGG CCUG	New Finding	DS639501	298055 to 298036	minus	CAGGTCTGGATGAGTGCCAATGTT GCGTAAATGCTGAATATTGCACTC GTCCCGGCCTT	-29.40	59	50.8	-0.98
rmi-miR-5317a	UUUCUUGU CUCUGUGU CGUCGUUU	New Finding	DS677713	284899 to 284882	minus	GTCTGCAAAAAACGAGCAAGCGC TGATTGGGCCGTCTCTGTGTCGTC GTTTCCTCAGGTGCGGAG	-19.10	65	56.92	-0.52
dme-miR-125	UCCCUGAG ACCCUAACU UGUGA	New Finding	DS978597	217092 to 217071	minus	CCCCACTCCCTGAGACCCTAACTT GTGATGCGTTCTGCGCCGCCTTCC CAGGTTAGATTCTCAGGCCCTGGG T	-32.10	73	60.27	-0.73
isc-miR-10	UACCCUGU AGAUCCGA AUUUGU	MI001226 2	DS891538	2780819 to 2780840	plus	TCTACATCTACCCTGTAGATCCGA ATTTGTCTGCAACAAGACTACAAA TTCGGTTCTAGAGAGGTTTGTGTG GT	-31.10	74	50	-0.84
isc-miR-133	UUGGUCCCC UUCAACCA GCUGU	MI001226 6	DS613658	228784 to 228763	minus	ACCAACTTTAGCTGGCTGAAGCCG GGCCAAATCGTCATAATTCCCATG AAAAGGAATCTCAATACATTTGGT CCCCTTCAACCAGCTGTGGTTGGC	-42.00	96	47.92	-0.91

miRNA	mature Sequence	miRBase Accession Number	*Ixodes* genome ID	Blast Hit info	strand	Pre-Mature Seq	MFE	bp	G+C %	MFEI
isc-miR-124	UAAGGCACGCGGUGAAUGCCAAG	MI0012265	DS840700	85204 to 85226	plus	GCTCTCCGTATTCACTGCGCGCCTTGATGTGCCCAAACGTATCATAAGGCACGCGGTGAATGCCAAGAGG	-36.20	70	55.71	-0.93
isc-miR-5310	UGUAGUCUGGCAGAAACGUCG	MI0018490	DS649088	485403 to 485383	minus	GCGGCGTTTGGTCGGACTACACCGTGAGGGTGCTCCTGGAGTCGAGGTGTAGTCTGGCAGAAACGTCGG	-37.40	69	62.32	-0.87
isc-miR-305	AUUGUACUUCAUCAGGUGCUCUG	MI0016444	DS945001	228337 to 228315	minus	AGTTAATTGTACTTCATCAGGTGCTCTGGAGTTCGGTTCGCACAACCAGGCATCTTTTGGAGTGCAAATGATA	-29.30	73	43.84	-0.92
isc-let-7	UGAGGUAGUAGGUUGUAUAGU	MI0012260	No hits							

Isc-Ixodes scapularis;
rmi-Rhipicephalus microplus;
hlo-Haemaphysalis longicornis;
dme-Drosophila melanogaster;
prd-Panagrellus redivivus
