# Supplementary material for: Repression of tick microRNA-133 induces organic anion transporting polypeptide expression critical for Anaplasma phagocytophilum survival in the vector and transmission to the vertebrate host
Source: PLoS Genet. 2020 Jul 2;16(7):e1008856. doi: 10.1371/journal.pgen.1008856 (PMC7331985; doi:10.1371/journal.pgen.1008856)
Supplement: S3 Table — Table shows P value from one-way ANOVA analysis for all the data shown in Figs 5, 7A and 7B. (PDF) [file pgen.1008856.s010.pdf]

S3 Table. ANOVA analysis for the data presented in Figures 5, 7A and 7B

Figures	Data comparison	One way ANOVA P value
Figure 5A	Control - miR-133 mimic	< 0.0001
	Control - miR-133 Inhibitor	
	miR-133 mimic - miR-133 Inhibitor	
Figure 5B	Control - miR-133 mimic	0.5511
	Control - miR-133 Inhibitor	
	miR-133 mimic - miR-133 Inhibitor	
Figure 5C	Control - miR-133 mimic	0.0013
	Control - miR-133 Inhibitor	
	miR-133 mimic - miR-133 Inhibitor	
Figure 5D	Control - miR-133 mimic	0.034
	Control - miR-133 Inhibitor	
	miR-133 mimic - miR-133 Inhibitor	
Figure 5E	Control - pre miR-133 mimic	0.0021
	Control - miR-133 Inhibitor	
	pre miR-133 mimic - miR-133 Inhibitor	
Figure 5F	Control - pre miR-133 mimic	0.0476
	Control - miR-133 Inhibitor	
	pre miR-133 mimic - miR-133 Inhibitor	
Figure 5G	Control - pre miR-133 mimic	0.0008
	Control - miR-133 Inhibitor	
	pre miR-133 mimic - miR-133 Inhibitor	
Figure 7A	Control - pre miR-133 mimic	0.0002
	Control - miR-133 Inhibitor	
	pre miR-133 mimic - miR-133 Inhibitor	
Figure 7B	Control - pre miR-133 mimic	0.0679
	Control - miR-133 Inhibitor	
	pre miR-133 mimic - miR-133 Inhibitor	
